# Eating Disorders in Individuals With Celiac Disease and Their Biological First-Degree Relatives

**DOI:** 10.1001/jamanetworkopen.2026.43815

**Published:** 2026-11-12

**Authors:** Jialu Yao, Jiangwei Sun, David Bergman, Karl Mårild, Shengxin Liu, Peter H. R. Green, Mariel Arvizu, Cynthia M. Bulik, Benjamin Lebwohl, Jonas F. Ludvigsson

**Affiliations:** 1 Department of Medical Epidemiology and Biostatistics, Karolinska Institutet, Stockholm, Sweden; 2 School of Public Health (Shenzhen), Sun Yat-Sen University, Shenzhen, Guangdong, China; 3 Department of Pediatrics, Institute of Clinical Sciences, Sahlgrenska Academy, Gothenburg, Sweden; 4 Department of Pediatrics, Queen Silvia Children’s Hospital, Gothenburg, Sweden; 5 Department of Medicine, Celiac Disease Center, Columbia University Irving Medical Center, New York, New York; 6 Takeda Development Center Americas, Inc., Cambridge, Massachusetts; 7 Department of Psychiatry, School of Medicine, University of North Carolina at Chapel Hill; 8 Department of Nutrition, Gillings School of Global Public Health, University of North Carolina at Chapel Hill; 9 Department of Epidemiology, Mailman School of Public Health, Columbia University, New York, New York; 10 Department of Pediatrics, Orebro University Hospital, Orebro, Sweden; 11 Department of Medicine, Columbia University College of Physicians and Surgeons, New York, New York

## Abstract

**Importance:**

Patients with celiac disease (CD) may face elevated risk of eating disorders, potentially due to genetic susceptibility and psychosocial factors of a gluten-free diet. However, up-to-date population-based evidence is scarce, and whether any excess risk extends to biological first-degree relatives (bFDRs) remains unclear.

**Objective:**

To investigate the risk of eating disorders in patients with CD and their bFDRs, including parents, full siblings, and offspring.

**Design, Setting, and Participants:**

A nationwide multigenerational cohort study of patients with celiac disease in Sweden, observed from January 2002 to December 2023. Data were analyzed from December 2025 to August 2026. From Sweden’s 28 histopathology departments, patients with biopsy-confirmed CD diagnosed 2002 to 2023 were identified. Each patient was matched with fewer than or equal to 5 general population reference individuals; bFDRs of patients with CD and reference individuals were also identified.

**Main Outcomes and Measures:**

The primary outcome was any eating disorder. The adjusted hazard ratio (aHR) for eating disorders diagnosed at age 6 years or older were estimated using Cox regression and flexible parametric survival models for the overall and time-varying estimates, respectively.

**Results:**

This study included 37 853 patients with CD (23 704 [62.6%] female; median [IQR] age at index date 28.7 [11.7-53.0] years ) and 185 159 reference individuals. Over 10.9 years median follow-up (IQR, 5.9-15.8 years), 485 patients with CD and 1578 reference individuals were diagnosed with any eating disorder. CD was associated with an increased hazard for eating disorders (aHR, 1.39; 95% CI,1.27-1.53), most apparent during the first 5 years of follow-up. Over 20 years of follow-up, 1 extra eating disorder case was expected per 222 patients with CD. In contrast, there was no hazard elevation for any eating disorder among CD-bFDRs (53 435 parents: aHR, 0.82 [0.61-1.12]; 48 743 siblings: aHR, 1.11 [0.99–1.24]; 38 695 offspring: aHR, 1.05 [0.92-1.20]). Sensitivity analyses mitigating information bias and residual confounding largely aligned with main findings.

**Conclusions and Relevance:**

In this cohort study of patients with CD, a moderately increased eating disorders risk particularly during the first 5 years of follow-up was observed, although the absolute risk was low. This increased risk did not extend to bFDRs of patients with CD. Clinical vigilance toward disordered eating is important among patients with newly diagnosed CD.

## Key Points

**Question:**

Are patients with celiac disease (CD) and their biological first-degree relatives (bFDRs) at increased risk of incident eating disorders?

**Findings:**

This cohort study of 37 853 multigenerational patients in Sweden with CD observed a moderately, but statistically significantly increased hazard for eating disorders in patients with CD, which was most prominent during the first 5 years of follow-up, although the absolute risk was low. Associations with any eating disorder were statistically nonsignificant among bFDRs of patients with CD.

**Meaning:**

These findings suggest that in newly diagnosed patients with CD, clinical vigilance for signs of disordered eating is important.

## Teaser

This cohort study investigates the risk of eating disorders in patients with celiac disease and their biological first-degree relatives.

## Introduction

Celiac disease (CD) affects about 1% of people worldwide.^1^ The condition arises from loss of immunological tolerance to gluten in genetically predisposed individuals, leading to intestinal inflammation and villus atrophy (VA).^1^ A strict gluten-free diet (GFD) remains the only treatment,^2^ but can be burdensome and stressful.^1,3^

Hypervigilance over food and anthropometrics in CD management may foster maladaptive eating behaviors.^3,4,5^ A positive association between CD and eating disorders has been consistently reported (eTable 1 in Supplement 1). However, most population-based evidence captures diagnoses that predate the *Diagnostic and Statistical Manual of Mental Disorders* (Fifth Edition),^6,7,8,9^ or excluded individuals with other psychiatric comorbidities.^10,11,12^

The impact of CD on lifestyles may extend to the patient’s family. A study in 40 children with CD reported more frequent snacking and mindless eating among both affected children and their parents following GFD initiation,^13^ suggesting a heightened risk for disordered eating.^14^ However, the link between CD and eating disorders among family members remains understudied and inconsistent.^12,15,16,17,18^

This Swedish nationwide multigenerational cohort study investigated the risk of incident eating disorders in patients with biopsy-confirmed CD compared with reference individuals from the general population, and in patients’ biological first-degree relatives (bFDRs, including parents, full siblings, and offspring) compared with the bFDRs of reference individuals. We hypothesized that both patients with CD and their bFDRs were at increased risk for developing eating disorders.

## Methods

### Data Sources

Histopathology data were retrieved from the nationwide Epidemiology Strengthened by Histopathology Reports in Sweden (ESPRESSO) cohort, which contains gastrointestinal reports from Sweden’s 28 pathology departments (1965 to 2023).^19,20^ Individuals from ESPRESSO were linked to nationwide health care and social insurance registers via unique personal identity numbers.^21^This study was approved by the Stockholm Ethics Review Board. Individual informed consent was waived as the study was register-based. Strengthening the Reporting of Observational Studies in Epidemiology (STROBE) reporting guideline was followed.^31^

The Swedish National Patient Register (NPR) collects specialized care data,^22,23^ with nationwide coverage on inpatient care since 1987 and outpatient care in 2001.^22^ Positive predictive values (PPVs) for diagnoses in the NPR were generally 80% to 95%.^22,23^ Both primary and secondary diagnoses were considered for outcomes and disease history. Education data (available for individuals 16 years of age or older since 1990) was obtained from the Swedish LISA database.^24^

### Patients With CD and Their Comparison Group

From ESPRESSO, we identified patients with CD indicated by duodenal or jejunal VA (Marsh 3; eTable 2 in Supplement 1) since 2002 (1 year after the nationwide coverage of specialized outpatient care) through 2023.^19^ The algorithm had a PPV of at least 95%.^25,26^

#### Matched Reference Individuals to Patients With CD

Up to 5 CD-free reference individuals were matched to each patient with CD from the Total Population Register (TPR) by age, sex, calendar year of CD diagnosis, and county of residence.^21^ Patients with CD and reference individuals were defined as index individuals.

The index date was the later date of the first duodenal or jejunal VA-indicating biopsy (or matching for reference individuals) or the individual’s sixth birthday (the age cutoff was selected to reduce potential misclassification of feeding disorders^27^). We excluded index individuals with detected chromosomal abnormalities, prior conditions with disordered eating behaviors (both outcomes and conditions secondary to other psychiatric conditions; eTable 3 in Supplement 1), emigration, or death up to the index date.

### Patients With Persistent VA and Mucosal Healing

We identified patients with CD who had follow-up biopsy 0.5 to 5.0 years after diagnosis, applying the same exclusion criteria as in the analysis between CD and reference individuals. Based on mucosal status, patients showing persistent VA (March 3) were compared with those with mucosal healing (Marsh 0 to 2). The index date was the later date of follow-up biopsy or the patient’s sixth birthday.

### Biological First-Degree Relatives of Patients With CD and Their Comparison Groups

From the Swedish Multi-Generation Register, we identified bFDRs (parents, full siblings, and offspring) of all index individuals, with the same exclusion applied.^21^ As individuals were not individually matched, covariate balance between comparison groups was inspected for a standardized mean difference of less than 0.1. The index date for bFDRs was the later of index individual’s CD diagnosis or matching, and the date when siblings or offspring turned 6 years.

### Outcome Ascertainment

The primary outcome was any eating disorder identified from the NPR (eTable 4 in Supplement 1).^22^ Secondary outcomes were anorexia nervosa (PPV, 74.7% for NPR-recorded cases)^28^ and other eating disorders, including bulimia nervosa (PPV, 83.6%)^28^ and unspecified eating disorders (PPV, 76.1%).^28^

### Follow-Up

We followed individuals from the index date until the earliest occurrence of each investigated outcome, emigration, death, or December 31, 2023. Reference individuals to CD were censored and reclassified as the exposed when diagnosed with CD. The same reclassification was applied to bFDRs of reference individuals when their index family member was diagnosed with CD (Figure 1).

**Figure 1.**
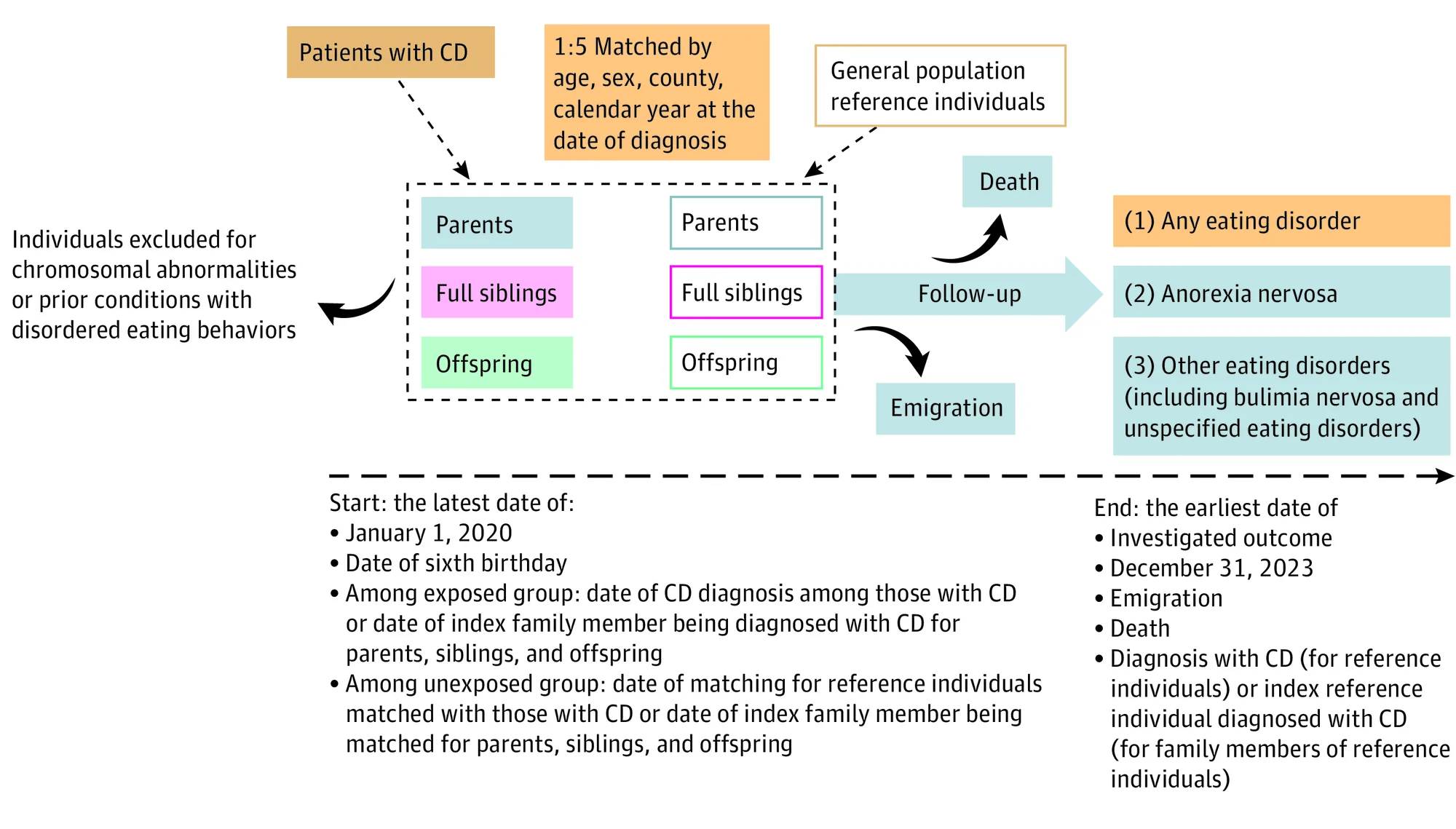
Study Design Diagram CD indicates celiac disease.

### Statistical Analyses

We reported absolute and relative risks with incidence rate (IR) differences and adjusted hazard ratios (aHRs), comparing patients with CD to reference individuals, or CD-bFDRs to reference individuals-bFDRs. The underlying time scale was time since index date.

Model 1 adjusted for age (continuous), sex, calendar year, and county of residence, (ie, adjusted for matching variables in the comparison between patients with CD and their reference individuals). Model 2 additionally adjusted for educational attainment (0 to 9 years, 10 to 12 years, at least 13 years, or missing; for individuals younger than 22 years, we used the highest education among the individual and his or her parents); country of birth (Nordic or others); number of specialized care visits 24 to 6 months before the index date (0, 1, 2 to 3, and at least 4); and separately for type 1 diabetes, inflammatory bowel disease, and juvenile idiopathic arthritis/rheumatoid arthritis ever before the index date (eTable 5 in Supplement 1). The comparison of patients with persistent VA vs those with mucosal healing was also adjusted for the duration of CD diagnosis before follow-up biopsy. In the analysis between patients with CD and reference individuals, a cluster-robust (sandwich) estimator controlled for intra-individual correlation.

When investigating eating disorders risk in CD-bFDRs, in addition to covariates in model 2, we adjusted for their index family member’s age, sex, and number of health care visits 24 to 6 months before the index date, and conditioned on a family identifier. The weighted average of multivariable-adjusted HRs over follow-up period were estimated using Cox proportional hazard models; flexible parametric models examined time-varying HRs and standardized cumulative incidence differences.^29^

For patients with CD and CD-bFDRs, subgroup analyses explored the risk of eating disorders by the following covariates of included individuals at the index date: sex; age; calendar period; educational attainment; any prior type 1 diabetes, inflammatory bowel disease, or juvenile idiopathic arthritis or rheumatoid arthritis; and prior psychiatric disorder or suicide attempt. Analyses among bFDRs were further stratified by age and sex of the index family member. Interaction effects were assessed by the Wald test given cluster-based variance estimation.

Sensitivity analyses were conducted. First, to further mitigate potential confounding from education, we restricted the analyses to those with available education data. Second, to minimize potential detection bias and reverse causation, we reran the analyses starting follow-up at 1 or 3 years after the index date. Third, we ascertained the outcomes requiring at least 2 or more records in the NPR. Fourth, we restricted the outcomes to those assigned in specialized psychiatric care. Fifth, we used attained age as the underlying time scale to more flexibly account for the potential confounding effect of age.^30^

Sixth and seventh, for patients with CD specifically, we additionally adjusted for their personal or familial (in any biological parents or full siblings) history of prior psychiatric disorder or suicide attempt. Eighth, we excluded bFDRs who developed CD at any time during follow-up to exclude the potential influence of CD per se.

Finally, to investigate the temporal relationship, we calculated the odds of eating disorders prior to CD diagnosis. This was conducted among individuals diagnosed or matched at greater than or at least 6 years of age, or among bFDRs, at greater than or at least 6 years at their index family member’s CD diagnosis or matching.

Analyses and visualizations were conducted between December 2025 to August 2026 using Stata version 18.1 (StataCorp), and R version 4.3.1 (R Project for Statistical Computing). A 2-sided *P* ≤ .05 was considered statistically significant. Findings for secondary outcomes and in subgroup and sensitivity analyses should be interpreted as exploratory because of potential type I error due to multiple comparisons.

## Results

### Eating Disorders in CD

This study involved 37 853 individuals with biopsy-confirmed CD (2002 to 2023; 23 704 [62.6%] female; median age at index date 28.7 years [IQR, 11.7-53.0 years]), matched with 185 159 reference individuals from the general population (115 916 [62.6%] female; median age 28.3 years [IQR, 11.6-52.4]) (baseline characteristics in Table 1; selection flowchart in eFigure in Supplement 1). During a median 10.9 years (IQR, 5.9-15.8 years) of follow-up, we observed incident eating disorders in 485 patients with CD and 1578 reference individuals (IR: 117 vs 78 per 100 000 person-years) (Figure 2; eTable 6 in Supplement 1). Hazard elevation was highest within the first 5 years of follow-up, declined rapidly to approximately 1.2 during 6 to 11 years, and became nonsignificant thereafter (Figure 3). This corresponded to 1 extra eating disorder case per 222 patients with CD during 0 to 20 years (eTable 7 in Supplement 1), and an aHR of 1.39 (95% CI, 1.27-1.53) throughout follow-up. Increased hazards were observed for both anorexia nervosa (aHR, 1.44; 95% CI, 1.24-1.67) and other eating disorders (aHR, 1.39; 95% CI, 1.25-1.54). However, among other eating disorders, associations were only significant for unspecified eating disorders (aHR, 1.45; 95% CI, 1.30-1.61) but not bulimia nervosa (aHR, 1.23; 95% CI, 0.95-1.59) (eTable 6 in Supplement 1).

**Table 1.** Characteristics of Patients With CD and Their Matched Reference Individuals

Characteristics	Individuals, No. (%)	
	Patients with CD (n = 37 853)	Reference individuals (n = 185 159)
Age at index date, y^a^		
Mean (SD)	33.7 (23.8)	33.4 (23.6)
Median (IQR)	28.7 (11.7-53.0)	28.3 (11.6-52.4)
6 to <10	7114 (18.8)	35 058 (18.9)
10 to <18	6107 (16.1)	30 443 (16.4)
18 to <30	5851 (15.5)	28 560 (15.4)
≥30	18 781 (49.6)	91 098 (49.2)
Female	23 704 (62.6)	115 916 (62.6)
Born in Nordic countries^b^	35 400 (93.5)	162 150 (87.6)
Calendar period at index date		
2002 to 2012	21 390 (56.5)	104 746 (56.6)
2013 to 2023	16 463 (43.5)	80 413 (43.4)
Educational attainment, y		
0 to 9	4306 (11.4)	23 104 (12.5)
10 to 12	13 800 (36.5)	69 566 (37.6)
≥13	19 338 (51.1)	89 643 (48.4)
Missing	409 (1.1)	2846 (1.5)
No. of health care visits^c^		
0	17 178 (45.4)	109 668 (59.2)
1	6814 (18.0)	30 760 (16.6)
2 to 3	6748 (17.8)	25 229 (13.6)
≥4	7113 (18.8)	19 502 (10.5)
History of any following autoimmune diseases^d^		
Type 1 diabetes	1823 (4.8)	1707 (0.9)
Inflammatory bowel disease	910 (2.4)	311 (0.2)
Juvenile arthritis or rheumatoid arthritis	367 (1.0)	1073 (0.6)
History of psychiatric disorder or suicide attempts^d^	3753 (9.9)	15785 (8.5)
Follow-up time, y		
Mean (SD)	10.9 (5.9)	10.9 (5.9)
Median (IQR)	10.9 (5.9 - 15.8)	11.0 (5.9 - 15.8)
0 to 0.9	1061 (2.8)	4637 (2.5)
1.0 to 4.9	6797 (18.0)	33647 (18.2)
5.0 to 9.9	9299 (24.6)	45417 (24.5)
10.0 to 14.9	9778 (25.8)	47857 (25.8)
≥15.0	10918 (28.8)	53601 (28.9)
No. ( of all included individuals)	485 (1.3)	1578 (0.9)
Among those diagnoses with any eating disorder, age at diagnosis, y		
Mean (SD)	20.1 (7.7)	20.1 (8.7)
Median (IQR)	17.7 (15.1-22.7)	17.6 (15.2-22.2)
6 to <18	208 (42.9)	695 (44.0)
18 to <30	220 (45.4)	708 (44.9)
≥30	57 (11.8)	175 (11.1)

Abbreviation: CD, celiac disease.

^a^ Date of CD-indicative biopsy for patients with CD and date of selection for reference individuals.

^b^ Nordic countries: Sweden, Denmark, Finland, Norway, and Iceland.

^c^ Between 2 years and 6 months before the index date.

^d^ Codes for disease histories are listed in eTable 5 in Supplement 1.

**Figure 2.**
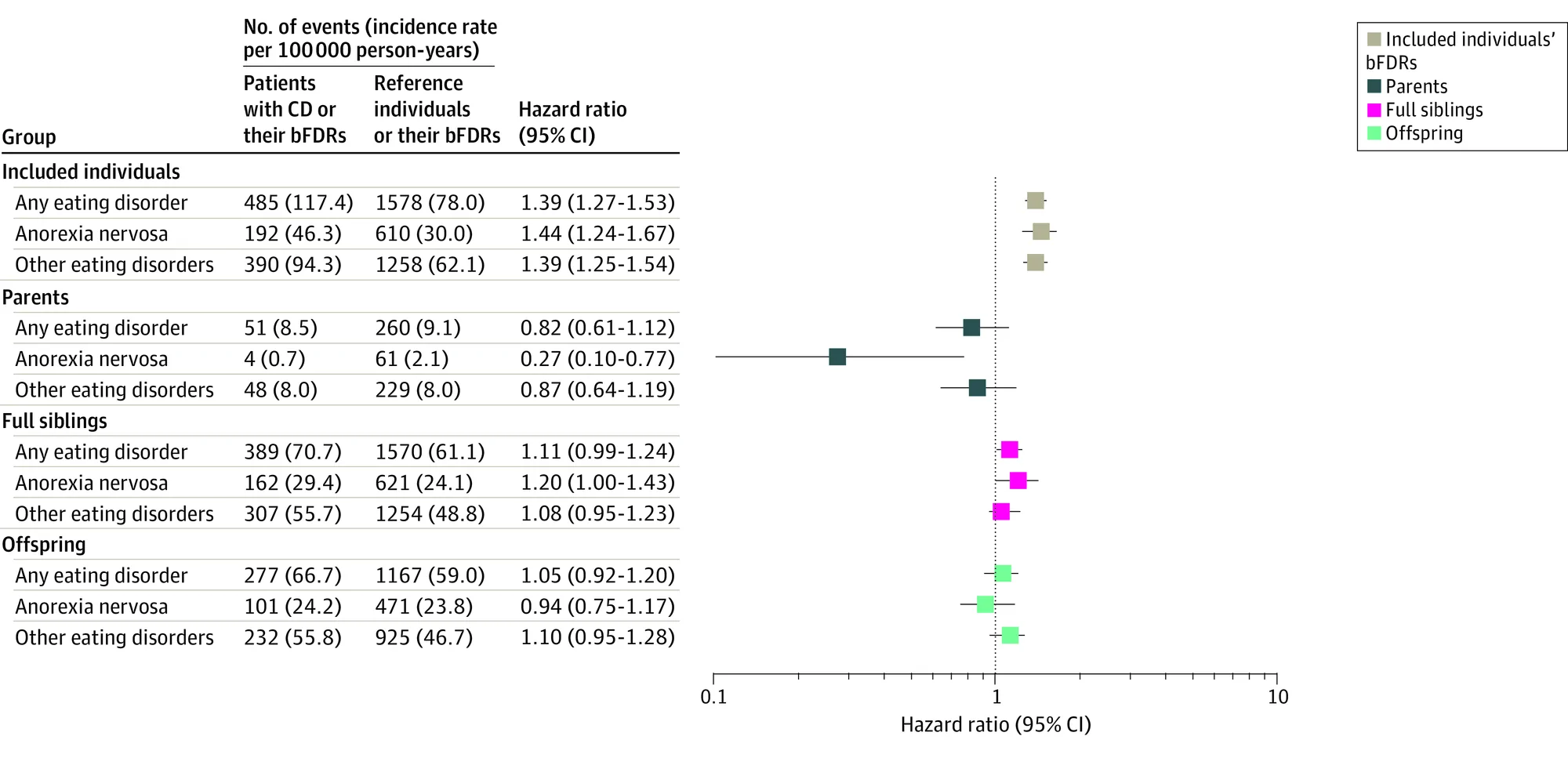
Dot Plot of Incident Eating Disorders in Patients With Celiac Disease, Their Matched Reference Individuals, and Both Groups’ Biological First-Degree Relatives The hazard ratio was estimated with the flexible parametric survival model based on model 2. bFDR indicates biological first-degree relative; CD indicates celiac disease.

**Figure 3.**
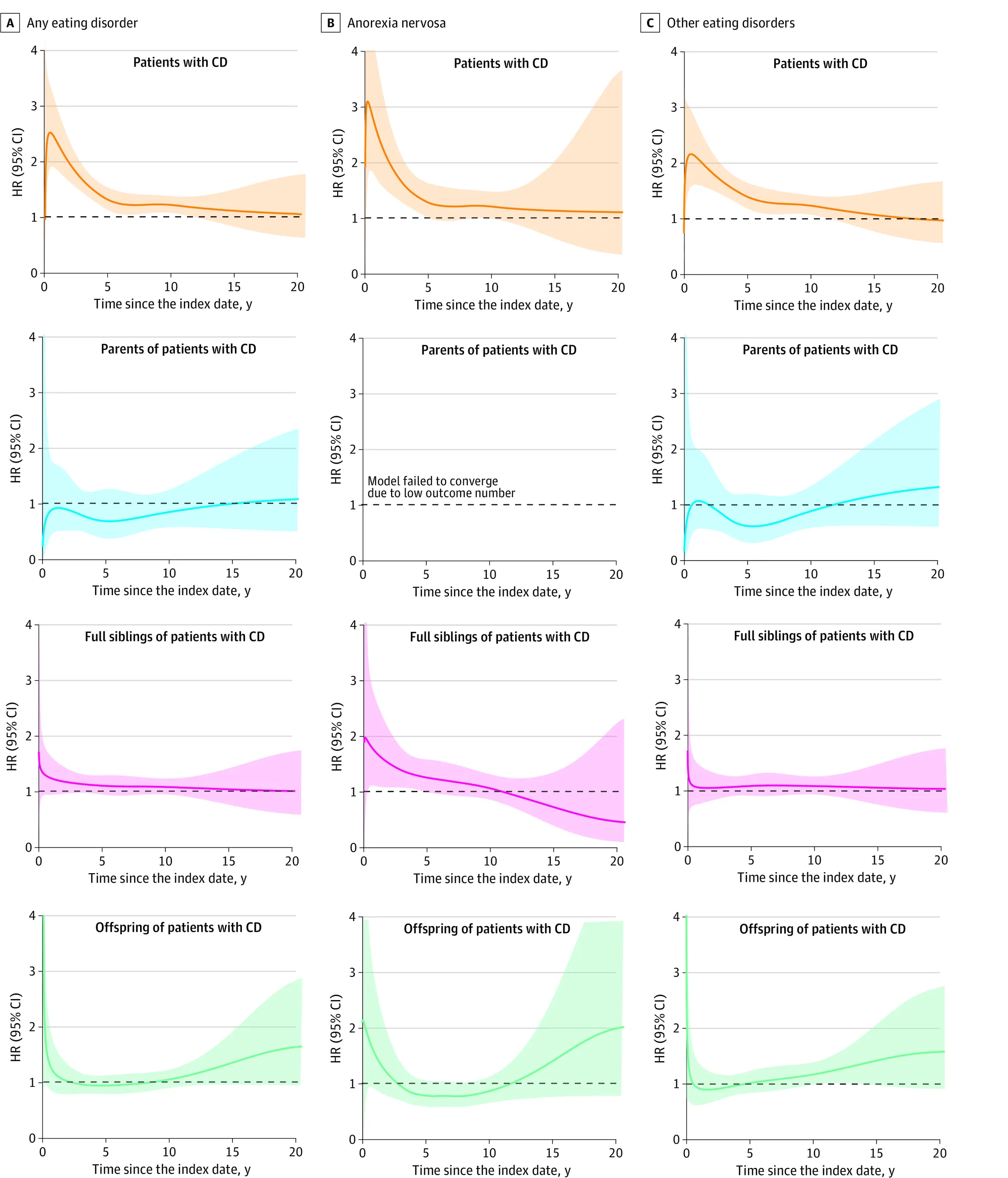
Line Graphs of Hazard Ratio for Eating Disorders in Patients With Celiac Disease Hazard ratio (HR) for eating disorders in patients with celiac disease (CD) compared with their matched reference individuals (lines in orange); and in biological first-degree relatives of patients compared with those of their reference individuals (lines in blue for parents, pink for full siblings, and green for offspring), with 95% CI (in shaded areas). The HRs were estimated with the flexible parametric survival model while being adjusted for covariates in model 2.

The positive association existed across sex, calendar period, and psychiatric disorder or suicide attempt history, but was restricted to individuals diagnosed at 10 years of age or older and those with at least 10 years of education (eTable 8 in Supplement 1). While IR and its excess were greater among female patients with CD, male patients had a higher relative hazard for any eating disorder (female patients: aHR, 1.35; 95% CI,1.23-1.49; and male patients: aHR, 2.30; 95% CI, 1.60 to 3.30; *P = *.05). Having psychiatric disorder or suicide attempt before the index date was associated with a near 5-fold hazard elevation (aHR, 4.80; 95% CI, 3.26-7.07; *P* < .001).

### Sensitivity Analyses of Eating Disorders in CD

CD remained positively associated with any eating disorder, anorexia nervosa, and other eating disorders when restricting analyses to individuals with education data, applying stricter outcome definitions, or excluding first 1 or 3 years of follow-up. Results remained largely unchanged when additionally adjusting for personal or familial history of psychiatric disorder or suicide attempt, or using attained age as underlying time scale (eTable 9 in Supplement 1). Eating disorders were also more frequent in patients with CD before their diagnosis (aOR, 1.30; 95% CI, 1.12-1.52 for any eating disorder) (eTable 10 in Supplement 1); although the excess was nonsignificant for diagnoses more than 1 year prior.

### Eating Disorders Risk by Mucosal Healing Status

Patients with persistent VA (n = 2344, 25.6% of patients with follow-up biopsy) were more commonly older (age at least 30 years: 1845 [78.7%] vs 3932 [57.8%]; *P* < .001), had lower female prevalence (1389 [59.3%] vs 4507 [66.3%]; *P* < .001), and lower education attainment (0 to 9 years: 449 [19.2%] vs 666 [9.8%]; *P* < .001) than those with mucosal healing (eTable 11 in Supplement 1). Eating disorders developed in 10 patients with persistent VA compared with 76 patients with mucosal healing (IR: 41 vs 103 per 100 000 person-years), translating to an aHR of 0.65 (95% CI, 0.33-1.28) (eTable 12 in Supplement 1).

### Eating Disorders in bFDRs of CD

We identified 53 435 parents (27 849 [52.1%] female; median [IQR] age at index date, 48.4 years [39.5-61.2 years]), 48 743 full siblings (23 933 [49.1%] female; median [IQR] age at index date, 26.8 years [11.8-50.0 years]), and 38 695 offspring (18 713 [48.4%] female; median [IQR] age at index date, 20.5 years [6.4-37.8 years]) of index patients with CD, who were compared with 254 596 parents, 229 226 siblings, and 186 249 offspring of index reference individuals (Table 2). Eating disorder incidence was similar between bFDRs of patients with CD vs reference individuals: parents (n = 51 vs 260; IR, 8.5 vs 9.1 per 100 000 person-years); siblings (n = 389 vs 1570; IR, 71 vs 61), and offspring (n = 277 vs 1167; IR, 67 vs 59). Neither hazard nor cumulative incidence of any eating disorder was increased in any CD-bFDR type, including parents (aHR, 0.82; 95% CI, 0.61-1.12), siblings (aHR, 1.11; 95% CI, 0.99-1.24), or offspring (aHR, 1.05; 95% CI, 0.92-1.20) ( Figure 2; eTable 13 in Supplement 1, standardized cumulative incidence difference in eTable 14 in Supplement 1). For anorexia nervosa specifically, a decreased hazard was observed among parents of individuals with CD although this observation was statistically underpowered, and based on only few observations in this subgroup. Among siblings of individuals with CD. Among CD-siblings, the hazard for anorexia nervosa was modestly increased (aHR, 1.20; 95% CI, 1.00-1.43), with statistical significance confined to the first 5 years after index family members’ CD diagnosis (Figure 3). Associations with bulimia nervosa or unspecified eating disorders were nonsignificant among CD-bFDRs.

**Table 2.** Characteristics of bFDRs of Patients With CD and Their Matched Reference Individuals, Stratified by Family Relationships

Characteristic	Parents of			Full siblings of			Offspring of		
	Patients with CD, No. (%) (n = 53 435)	Reference individuals, No. (%) (n = 254596)	SMD	Patients with CD, No. (%) (n = 48 743)	Reference individuals, No. (%) (n = 229 226)	SMD	Patients with CD, No. (%) (n = 38 695)	Reference individuals, No. (%) (n = 186 249)	SMD
No.	53 435	254 596	NA	48 743	229 226	NA	38 695	186 249	NA
Age at index date, y^a^			0.03			0.05			0.01
Mean (SD)	51.3 (15.2)	50.8 (15.3)	NA	31.8 (21.9)	30.7 (21.8)	NA	23.8 (16.5)	23.7 (16.3)	NA
Median (IQR)	48.4 (39.5-61.2)	47.7 (39.2-60.6)	NA	26.8 (11.8-50.0)	24.7 (11.1-48.9)	NA	20.5 (6.4-37.8)	20.5 (6.2-37.4)	NA
6 to <10	0 (0)	0 (0.0)	NA	9276 (19.0)	46 245 (20.2)	NA	11 728 (30.3)	56 186 (30.2)	NA
10 to <18	0 (0)	4 (0.0)	NA	7874 (16.2)	39 665 (17.3)	NA	5509 (14.2)	26 389 (14.2)	NA
18 to <30	1456 (2.7)	7833 (3.1)	NA	8565 (17.6)	41 236 (18.0)	NA	6849 (17.7)	34 245 (18.4)	NA
≥30	51 979 (97.3)	246 759 (96.9)	NA	23 028 (47.2)	102 080 (44.5)	NA	14 609 (37.8)	69 429 (37.3)	NA
Female	27 849 (52.1)	133 482 (52.4)	0.01	23 933 (49.1)	112 124 (48.9)	0.00	18 713 (48.4)	90 622 (48.7)	0.01
Born in Nordic countries^b^	48 822 (91.4)	212 368 (83.4)	0.24	47 041 (96.5)	214 621 (93.6)	0.13	37 289 (96.4)	176 823 (94.9)	0.07
Calendar period at index date									
2002-2012	31 156 (58.3)	150 105 (59.0)	0.01	27 046 (55.5)	126 774 (55.3)	0.00	19 347 (50.0)	91 596 (49.2)	0.02
2013-2023	22 279 (41.7)	104 491 (41.0)		21 697 (44.5)	102 452 (44.7)		19 348 (50.0)	94 653 (50.8)	
Educational attainment, y									
0 to 9	9511 (17.8)	50 145 (19.7)	0.07	5102 (10.5)	25 915 (11.3)	0.04	3045 (7.9)	16 629 (8.9)	0.09
10 to 12	24 044 (45.0)	114 506 (45.0)		18 916 (38.8)	91 524 (39.9)		13 258 (34.3)	64 617 (34.7)	
≥13	19 146 (35.8)	85 123 (33.4)		24 479 (50.2)	110 522 (48.2)		15 569 (40.2)	68 105 (36.6)	
Missing	734 (1.4)	4822 (1.9)		246 (0.5)	1265 (0.6)		6823 (17.6)	36 898 (19.8)	
No. of health care visits^c^									
0	27 844 (52.1)	140 973 (55.4)	0.07	27 676 (56.8)	140 874 (61.5)	0.11	22 892 (59.2)	117 676 (63.2)	0.09
1	9093 (17.0)	42 468 (16.7)		8141 (16.7)	36 907 (16.1)		6581 (17.0)	30 254 (16.2)	
2 to 3	8592 (16.1)	37 857 (14.9)		7060 (14.5)	29 266 (12.8)		5349 (13.8)	23 065 (12.4)	
≥4	7906 (14.8)	33 298 (13.1)		5866 (12.0)	22 179 (9.7)		3873 (10.0)	15 254 (8.2)	
History of any following autoimmune diseases^d^									
Type 1 diabetes	1076 (2.0)	3341 (1.3)	0.05	792 (1.6)	2002 (0.9)	0.07	494 (1.3)	1150 (0.6)	0.07
Inflammatory bowel disease	898 (1.7)	2962 (1.2)	0.04	526 (1.1)	1615 (0.7)	0.04	319 (0.8)	1161 (0.6)	0.02
Juvenile arthritis or rheumatoid arthritis	616 (1.2)	2200 (0.9)	0.03	348 (0.7)	1313 (0.6)	0.02	157 (0.4)	587 (0.3)	0.02
History of psychiatric disorder or suicide attempts^d^	5678 (10.6)	27 946 (11.0)	0.01	4147 (8.5)	18 943 (8.3)	0.01	2836 (7.3)	13 634 (7.3)	0.00
Follow-up time, years									
Mean (SD)	11.2 (6.0)	11.2 (6.0)	NA	11.3 (5.9)	11.2 (5.9)	NA	10.7 (6.2)	10.6 (6.2)	NA
Median (IQR)	11.4 (6.1-16.3)	11.3 (6.0-16.3)		11.3 (6.3-16.2)	11.3 (6.2-16.1)		10.5 (5.2-16.1)	10.3 (5.2-15.9)	
0 to 0.9	1087 (2.0)	5634 (2.2)	NA	963 (2.0)	4768 (2.1)	NA	1366 (3.5)	6912 (3.7)	NA
1.0 to 4.9	9540 (17.9)	46 249 (18.2)	NA	8211 (16.8)	39 793 (17.4)	NA	7751 (20.0)	38 033 (20.4)	NA
5.0 to 9.9	12 533 (23.5)	59 206 (23.3)	NA	11 980 (24.6)	55 662 (24.3)	NA	9406 (24.3)	45 596 (24.5)	NA
10.0 to 14.9	13 756 (25.7)	64 550 (25.4)	NA	12 561 (25.8)	59 126 (25.8)	NA	8748 (22.6)	41 926 (22.5)	NA
≥15.0	16 519 (30.9)	78 957 (31.0)	NA	15 028 (30.8)	69 877 (30.5)	NA	11 424 (29.5)	53 782 (28.9)	NA
Diagnosed with any eating disorder									
No. (% of all included individuals)	51 (0.1)	260 (0.1)	NA	389 (0.8)	1570 (0.7)	NA	277 (0.7)	1167 (0.6)	NA
Among those diagnosed with eating disorder, age at diagnosis, y									
Mean (SD)	42.9 (10.9)	42.9 (11.6)	NA	19.9 (8.0)	20.0 (8.0)	NA	20.3 (8.1)	20.2 (8.4)	NA
Median (IQR)	42.3 (36.8-47.6)	41.0 (35.7-48.8)	NA	17.3 (15.2-22.7)	17.6 (15.1-22.3)	NA	17.7 (14.5-24.7)	17.4 (14.7-22.8)	NA
6 to <18	0	0	NA	177 (45.5)	703 (44.8)	NA	122 (44.0)	552 (47.3)	NA
18 to <30	2 (3.9)	23 (8.8)	NA	171 (44.0)	704 (44.8)	NA	115 (41.5)	464 (39.8)	NA
≥30	49 (96.1)	237 (91.2)	NA	41 (10.5)	163 (10.4)	NA	40 (14.4)	151 (12.9)	NA

Abbreviations: bFDR, biological first-degree relative; CD, celiac disease; NA, not applicable; SMD, standardized mean difference.

^a^ Date of CD-indicative biopsy for patients with CD and date of selection for reference individuals.

^b^ Nordic countries: Sweden, Denmark, Finland, Norway, and Iceland.

^c^ Between 2 years and 6 months before the index date.

^d^ Codes for disease histories are listed in eTable 5 in Supplement 1.

Subgroup results were mostly concordant with the main findings among CD-bFDRs (eTable 15 in Supplement 1), with statistically significantly positive associations in subgroups such as siblings aged 10 to younger than 18 years at index date, siblings who had a prior psychiatric disorder or suicide attempt, and offspring whose index parent’s age at CD diagnosis was 18 to younger than 30 years. The associations were not significant across most sensitivity analyses among bFDRs of patients with CD (eTable 16 in Supplement 1); there were also null associations with prior eating disorders (eTable 17 in Supplement 1).

## Discussion

This nationwide cohort included more than 37 000 patients with biopsy-confirmed CD and more than 140 000 CD-bFDRs from 2002 through 2023. CD was associated with a moderately increased risk for eating disorders (aHR estimate, 1.39), with peak-risk period being the first 5 years of follow-up. Subgroup results showed a higher HR estimate in males (aHR, 2.30) and in those with prior psychiatric disorder or suicide attempt (aHR, 4.80). By eating disorder phenotype, the hazard in patients with CD was increased for anorexia nervosa and other unspecified eating disorders, but not bulimia nervosa.

In contrast, among bFDRs, only full siblings of newly diagnosed patients showed a modest (aHR, 1.20) and short-term increase (within 5 years) in anorexia nervosa risk, while no associations were observed across other CD-bFDRs types or for other investigated eating disorders. Our findings aligned closely with those derived earlier from the Swedish population (eTable 1 in Supplement 1).^6,7,11,12^ For any eating disorder and its phenotypes, the temporal pattern resembled that of Hedman et al,^6^ who noted that hazard elevations were highest up to 4 years after CD diagnosis (within 5 years in our study). Our estimate for any eating disorder in childhood-onset CD (1.33; eTable 8 in Supplement 1) was close to prior estimate of 1.34 (1.40 up until adulthood).^11,12^ For anorexia nervosa, Mårild et al^7^ (1987 to 2009) reported an aHR of 1.42 in females with CD, same as our female-specific estimate (eTable 6 in Supplement 1 footnotes). Meanwhile, studies from Canada, Denmark, and the UK reported a more apparent association, being 2.1 to 2.2 (in aHR)^10,32^ for any eating disorder and 2.46 (among females, in adjusted relative risk) for anorexia nervosa.^8^ These higher estimates may reflect a shorter follow-up (eg, mean 10.9 in ours vs 6.6 years in the Danish study^10^), or differences in study design (eg, controls also free from inflammatory bowel disease in the Canadian study^32^), data source (eg, inpatient CD cases in the UK study likely faced greater health needs^8^), or population characteristics (eg, sex, age, ancestry).

We also corroborated epidemiological evidence among CD-bFDRs,^12,18^ and extended observations to offspring. Taken together with the findings among parents by Nevriana et al,^18^ our results suggest that the null association between CD and parental eating disorders exists in both temporal directions. Although our null findings in siblings for overall eating disorders were consistent with Butwicka et al,^12^ interpretations may differ. The prior study followed up CD-free siblings from birth to age 18 years, thereby ruling out shared genetics and early environmental confounders that may have contributed to the positive association between CD and childhood-onset eating disorders.^12^ Our study, on the other hand, was additionally premised on the intrafamilial impact of CD-related dietary change.

The psychosocial burden of a GFD and its potentially adverse nutritional impact could contribute to the short to midterm increase in eating disorders risk among patients with CD. Weight gain may follow the adoption of a GFD, particularly when of poor nutritional quality,^3,4^ potentially fostering dietary anxiety and worsened eating behaviors.^3,4,14^ Hypervigilance to avoid gluten exposure may further worsen the challenging relationship with food, as suggested by the higher incidence of anxiety in patients with mucosal healing than those with persistent VA.^33^

Microbiota-mediated neuroinflammation is another plausible pathway. Impaired microbial fiber metabolism has been recently noted in CD including patients on a GFD, with their duodenal microbiota revealing depletion of fiber-degrading taxa.^34^ This parallels findings in eating disorders, in which reduced short-chain fatty acid production increases the blood-brain barrier permeability to inflammatory mediators.^35^ The resulting neuroinflammation has been speculated to disrupt the food reward system and elicit eating disorders.^35^ Although familial patterns alone did not support a genetic mediation,^15,16,17,36^ studies on genetic variants are necessary to determine shared genetic factors underlying CD and eating disorders.

The temporal proximity between CD and eating disorder diagnoses (Figure 2; eTable 10 in Supplement 1) suggested that overlapping symptoms, such as nutritional deficiency and altered stool consistency (associated with purging behaviors in eating disorders),^1,9^ may prompt incidental detection of 1 condition during diagnostic work-up or follow-up for the other, inflating the association. Dietitian involvement, particularly when managing pediatric CD cases, may also help identify signs of eating disorders.^37^

This study leveraged nationwide, prospectively updated registers from Sweden’s tax-funded health care and social insurance system,^24,38^ with mandatory registrations minimizing selection bias.^21^ The data enabled investigation of eating disorders risk during the *DSM-5 *era (2013 onward), reflecting current CD management and eating disorders diagnosis paradigms. Outcome misclassification was low, as PPVs were greater than or equal to 95% for biopsy-verified CD^25,26^ and 75% to 84% for eating disorder phenotypes.^28^

### Limitations

There were also limitations. First, detection and surveillance biases were implied since eating disorders were presumably more likely to be detected among patients with CD. However, the association persisted in lag-time analyses. Second, absence of primary care data may have resulted in missed eating disorder cases, although most eating disorder diagnoses in Sweden are assigned by specialists.^39^ Third, the registers lacked data on body measurements and the behavioral and psychological features of disordered eating. This prevented us from identifying potentially undetected eating disorder cases based on the DSM-5 criteria,^9^ and differentiating the risk by eating disorder severity and anorexia nervosa subtypes. Relatedly, we could only speculate about the impact of a GFD and mucosal status with little information on diet and low outcome numbers in patients with follow-up biopsy. Fourth, caution should be exercised when interpreting the results of some statistically underpowered analyses (eg, the comparison between patients with persistent VA and mucosal healing, the analysis for parental eating disorders, and certain subgroup analyses). Fifth, given genetic predispositions for both CD and eating disorders,^1,5^ the European-ancestry predominance of this cohort, and cross-cultural differences in diet and social norms, our findings should be extrapolated to other populations with caution. Additionally, our observations do not imply causality.

The marked short to midterm risk elevation for eating disorders after the index date (equivalent to CD diagnosis for those aged 6 years or older) underpins clinical vigilance toward disordered eating behaviors in newly diagnosed patients.^2,3^ Health care professionals and patient education materials should approach the GFD with prudence to avoid food-related anxiety. Detection of eating disorders in CD may be further complicated by overlapping symptoms, in addition to social stigma and underrecognition.^40^ Clinicians should therefore actively rule out eating disorders when symptoms among patients with CD (eg, nutritional deficiency, altered bowel habit) persist despite a GFD. Notably, patients with psychiatric comorbidities appeared to constitute a high-risk subgroup, highlighting the importance of coordinated care involving clinicians knowledgeable in both CD and eating disorders. While bFDRs of patients with CD did not show significant elevation in overall eating disorders risk, certain subgroups, such as siblings aged 10 to younger than 18 years and those with prior psychiatric disorder or suicide attempt may be more vulnerable to the ripple effect of their family member’s dietary restriction.^41^

## Conclusions

In this cohort study of patients with CD, there was a moderately elevated relative risk of eating disorders, particularly during the first 5 years of follow-up, although the absolute risk was low. However, this excess risk of eating disorders did not extend to biological parents, full siblings, or offspring of patients with CD.

## Notes

Supplement 1. eFigure. Individual selection flowchart eTable 1. Multi-centre or population-based evidence (published in 2016 onward) on eating disorders risk in CD eTable 2. SNOMED codes defining CD a eTable 3. Definitions of exclusion criteria eTable 4. Definitions of outcomes eTable 5. Definitions of comorbidities eTable 6. Incident eating disorders in patients with CD and their matched reference individuals eTable 7. Cumulative incidence difference (95%CI) of incident eating disorders during follow-up in individuals with CD, compared with their matched reference individuals eTable 8. Subgroup analyses of any incident eating disorder in patients with CD and their matched reference individuals eTable 9. Sensitivity analyses of incident eating disorders in patients with CD and their matched reference individuals eTable 10. Sensitivity analyses on eating disorders before the diagnosis of CD, among individuals diagnosed ≥6 years of age eTable 11. Characteristics of CD patients who had a follow-up biopsy between six months and five years after diagnosis, n (%) eTable 12. Incident eating disorders in CD patients who had persistent VA compared with those with mucosal healing eTable 13. Incident eating disorders in bFDRs of patients with CD and their matched reference individuals eTable 14. Cumulative incidence difference (95%CI) of incident eating disorders during follow-up in bFDRs of patients with CD and their matched reference individuals eTable 15. Subgroup analyses of any incident eating disorder in bFDRs of patients with CD and their matched reference individuals1 eTable 16. Sensitivity analyses of incident eating disorders in bFDRs of patients with CD and their matched reference individuals eTable 17. Sensitivity analyses on eating disorders in bFDRs of patients with CD before the index date, among individuals ≥6 years of age at their index family members’ CD diagnosis eReferences.

Supplement 2. Data Sharing Statement
