# Supplementary material for: Eating Disorders in Individuals With Celiac Disease and Their Biological First-Degree Relatives
Source: JAMA Netw Open. 2026 Nov 12;9(11):e2643815. doi: 10.1001/jamanetworkopen.2026.43815 (PMC13760515; doi:10.1001/jamanetworkopen.2026.43815)
Supplement: Supplementary file 1 [file jamanetwopen-e2643815-s001.pdf]

Supplemental Online Content

Yao J, Sun J, Bergman D, et al. Eating disorders in celiac disease and biological first-degree relatives. *JAMA Netw Open.* 2026;9(11):e2643815. doi:10.1001/jamanetworkopen.2026.43815

eFigure. Individual selection flowchart

eTable 1. Multi-centre or population-based evidence (published in 2016 onward) on eating disorders risk in CeD

eTable 2. SNOMED codes defining CeD a

eTable 3. Definitions of exclusion criteria

eTable 4. Definitions of outcomes

eTable 5. Definitions of comorbidities

eTable 6. Incident eating disorders in patients with CeD and their matched reference individuals

eTable 7. Cumulative incidence difference (95%CI) of incident eating disorders during follow-up in individuals with CeD, compared with their matched reference individuals

eTable 8. Subgroup analyses of any incident eating disorder in patients with CeD and their matched reference individuals

eTable 9. Sensitivity analyses of incident eating disorders in patients with CeD and their matched reference individuals

eTable 10. Sensitivity analyses on eating disorders before the diagnosis of CeD, among individuals diagnosed ≥6 years of age

eTable 11. Characteristics of CeD patients who had a follow-up biopsy between six months and five years after diagnosis, n (%)

© 2026 Yao J et al. *JAMA Network Open*.

eTable 12. Incident eating disorders in CeD patients who had persistent VA compared with those with mucosal healing

eTable 13. Incident eating disorders in bFDRs of patients with CeD and their matched reference individuals

eTable 14. Cumulative incidence difference (95%CI) of incident eating disorders during follow-up in bFDRs of patients with CeD and their matched reference individuals

eTable 15. Subgroup analyses of any incident eating disorder in bFDRs of patients with CeD and their matched reference individuals1

eTable 16. Sensitivity analyses of incident eating disorders in bFDRs of patients with CeD and their matched reference individuals

eTable 17. Sensitivity analyses on eating disorders in bFDRs of patients with CeD before the index date, among individuals ≥6 years of age at their index family members' CeD diagnosis

eReferences.

This supplemental material has been provided by the authors to give readers additional information about their work.

© 2026 Yao J et al. *JAMA Network Open*.

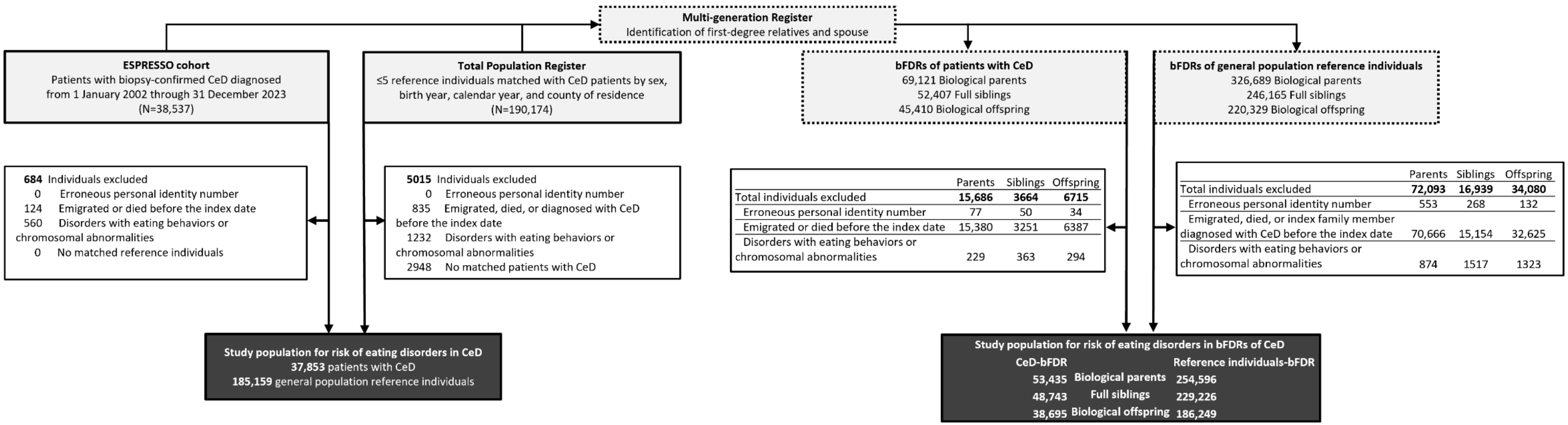

eFigure 1 Individual selection flowchart

bFDR: biological first-degree relative; CeD: celiac disease

© 2026 Yao J et al. *JAMA Network Open*.

eTable 1 Multi-centre or population-based evidence (published in 2016 onward) on eating disorders risk in CeD

**Current study**								
**First author, publication year, place, data source, study period, follow-up time**	**Sample size**	**Age at CeD diagnosis, years**	**Females, %**	**Number of outcomes in CeD**	**Incidence rate of outcome, per 100,000 person-years**	**First year of follow-up included?**	**Covariates**	**Main findings**
Yao, conducted in 2026, Sweden; Nationwide specialized care register plus a nationwide histopathology cohort; From 2002 through 2023; Median follow-up: 10.9 years (IQR: 5.9-15.8)	Biopsy-confirmed CeD: 37,853	Median: 28.7 years, Aged ≥6 years	62.6	Any eating disorder: 485<br><br>Anorexia nervosa: 192<br><br>Other eating disorders: 390: including Bulimia nervosa: 68 Unspecified eating disorders: 356	Any eating disorder (CeD vs. references, same below): 117 (vs. 78) Anorexia nervosa: 46 (vs. 30) Other eating disorders: 94 (vs. 62) Bulimia nervosa: 16 (vs. 12) Unspecified eating disorders: 86 (vs. 55)	Included, excluded in sensitivity analysis	(Model 1)<br>1. age<br>2. sex<br>3. county of residence<br>4. calendar year<br>+<br>(Model 2)<br>5. country of birth<br>6. education level<br>7. number of healthcare visits (between 6 and 24 months prior)<br>8. and separately for the history of type 1 diabetes, inflammatory bowel disease, and juvenile idiopathic arthritis /rheumatoid arthritis.	aHR for primary outcome in patients with CeD, based on Model 2 (same as below):<br>Any eating disorder: 1.39 (95%CI: 1.25 to 1.55)<br><br>aHRs for secondary outcomes:<br>Anorexia nervosa: 1.44 (1.21 to 1.71)<br>Other eating disorders: 1.39 (1.23 to 1.56), including:<br>Bulimia nervosa: 1.23 (0.93 to 1.64)<br>Unspecified eating disorders: 1.45 (1.28 to 1.64)
**Individual studies**								
**First author, publication year, place, data source, study period, follow-up time**	**Sample size (CeD)**	**Age at CeD diagnosis, years**	**Females, %**	**Number of outcomes in CeD**	**Incidence rate of outcome, per 100,000 person-years**	**First year of follow-up included?**	**Covariates**	**Main findings**
Subramanian L,[1] 2024, Ontario Canada; Regional health administrative data (inpatient discharge, care service claims, ambulatory, and day surgery datasets), inpatient discharge for mental care for outcomes; Cases identified between from July 1, 2002 to December 31, 2020 and followed until January 31, 2022; median follow-up 13.1 years (IQR 7.2-19.6) for any incident case with CeD or inflammatory bowel disease	14,718	Only individuals <50 years included, median (IQR) 32.0 years (22.0-40.0) for individuals with any of CeD or IBD	54.5 for individuals with any of CeD or IBD	50	40 (95%CI: 30-53) vs. 19 (14-25)<br><br>118 (84-166) vs. 46 (31-67) for cases diagnosed ≤18 years; 17 (10-28) vs. 11 (7-17) for cases diagnosed >18 years	Included	Matching variables<br>1. age<br>2. sex<br>3. health region residence<br>+ adjusted covariates in the Cox model:<br>4. rural residence<br>5. socioeconomic status by residence area	There was a 2-fold increased risk for any eating disorder in patients with CeD, however this hazard elevation was restricted to individuals diagnosed before adulthood<br>In the overall population: aHR=2.11 (95%CI: 1.41 to 3.16);<br>For cases diagnosed ≤18 years of age: aHR=2.59 (1.55 to 4.32);<br>For cases diagnosed >18 years of age: aHR=1.52 (0.78 to 2.96)
Hansen S,[2] 2023, Denmark; Nationwide out- and inpatient database; Cases identified January 1, 2000 to December 31, 2018; mean follow-up in CeD 6.6 years (=61275/9284)	9284	Mean (SD): 27 (21); Median (IQR): 21 (9-41)	65	56 (0.6%)	90 vs 40	Included, but excluded in sensitivity analysis	Birth date and sex (matching variables)	Patients with CeD were at higher hazard for eating disorder compared with matched references aHR 2.18 (1.63 to 2.92). Results were robust in sensitivity analyses that only take primary diagnoses as the outcome or exclude the first year of follow up.
Lebwohl B,[3]* 2021, Sweden; Nationwide histopathology and specialized care register;	19,186 (free from any preceding	All <18 years, Median (IQR): 5.3 years (1.7-10.9)	62.9	331 (1.7)	120 (110-130) vs. 90 (80-90)	Included	Matching variables<br>1. age<br>2. sex<br>3. county	There was a higher incidence rate (120 vs. 90 per 100,000 person-years) of any eating disorder in children with CeD.

© 2026 Yao J et al. *JAMA Network Open*.

1 January 1973 to 31 December 2016;<br>Mean follow-up 14.4 years (=277,015/19,186) for eating disorders	psychiatric disorder)						4. calendar period<br><br>Additionally adjusted for:<br>5. highest parental educational level	Children with CeD had a higher hazard for incident eating disorder (HR: 1.34, 1.18 to 1.51).<br><br>Children with CeD also had higher odds for prior eating disorders (n=95 in 19,583 CeD, OR: 3.10, 2.39 to 4.02); association remained in the sibling comparison. However, the risk increase was no longer signifcant among childhood-onset CeD who remained free from psychiatric disorders up until 18 years.
Alkhayyat M,[4] 2021, the US;<br>Multicenter outpatient and inpatient database across the US (IBM Explorys Inc.,);<br>1999 to 2020;<br>follow-up N/A for case-control study	112,340	<18 years 5.6%;<br>18-65 years 69.7%<br>>65 years 24.7%	75.8	15,600 (13.9%)	N/A	N/A	1. age<br>2. sex<br>3. race	Patients with CeD had higher odds of an eating disorder *history* compared with the references, aOR=15.84 (15.53 to 16.15)
Hedman A,[5]* 2019, Sweden;<br>Nationwide specialized care register;<br>Cases born between January 1, 1979 and December 31, 2005 and followed up until December 2013;<br>mean follow-up for associations in both directions: 13.2 years (=33,640,644/2,545,611)	Among the birth cohort, CeD: n= 19,730<br>Any eating disorder: n=26,454	0-35 years, Mean (SD) in CeD: Females: 11.83 (7.5); Males: 10.31 (6.9)	63.1	In CeD:<br>Any eating disorder: 396 (=375[6]+21[M]);<br>Anorexia nervosa: 195 (=188[F]+7[M]);<br>Other eating disorders: 315 (=300[F]+15[M]);<br><br>In patients with eating disorder:<br>CeD:<br>334 (=316[F]+18[M]) among any eating disorder; 165 (=159[F]+ 6[M]) among anorexia nervosa; 271 (=258[F]+ 13[M]) among;	Not reported	Included	Time-dependent exposure<br>1. Calendar-time<br>2. age<br>3. socioeconomic status (highest parental education)	Risk for any eating disorder was increased among both females (aHR=1.45 [1.28 to 1.63]) with CeD) and males (aHR=1.82 [1.07 to 3.08], n.s. after false discovery rate correction); the risk was limited to female CeD (but not males) for anorexia nervosa (aHR=1.50 [1.32 to 1.71]) and other eating disorders (aHR=1.47 [1.26 to 1.71]). Associations with bulimia nervosa were non-significant in both sexes.<br><br>Any eating disorder was associated with a higher risk for incident CeD for both females (aHR=1.72 [1.40 to 2.10]) and males (aHR=3.75 [1.79 to 7.88], n.s. after false discovery rate correction, Q-value=0.06); The positive association was also seen for other eating disorders (females: aHR=1.69 [1.32 to 2.17]) and males: aHR=3.79 ([1.58 to 9.12], n.s. after false discovery rate correction) but limited to females for anorexia nervosa (aHR=1.83 [1.40 to 2.39]).
Mårild K,[7]* 2017;<br>Nationwide histopathology and specialized care register;<br>from 1987 through 2009;<br>mean follow-up for both CeD and references: 10.9 years (=1,174,401/(17,959+89,379))	CeD (Marsh III): 17,959<br>Marsh I–II: 7455;<br>Marsh 0 but positive serology: 2307	Median (IQR):<br>CeD: 28 years (6-52),<br>Marsh I-II: 46 years (31-63),<br>Marsh 0 but positive CeD serology: 38 years (23-53)	100	CeD: 54<br>Marsh I-II: 9;<br>Marsh 0 but positive serology: 9	CeD: 27 (95%CI 21-36) vs. 18 (16-21);<br>Marsh I-II: 13 (7-25) vs. 6 (4-9);<br>Marsh 0 but positive serology: 47 (24-90) vs. 20 (13-31)	Included, but excluded in sensitivity analysis	Matching variables<br>1. age<br>2. sex<br>3. county<br>4. calendar period<br>Additionally adjust for<br>5. educational level (individuals' for adults and parents' for those <18 years)<br>6. socioeconomic status (occupational status according to the European Socioeconomic Classification system)<br>7. type 1 diabetes	Females with CeD had a higher hazard for anorexia nervosa (aHR=1.42 [1.04 to 1.94]), which became non-significant (aHR=1.29 [0.92 to 1.78]) when excluding the first year of follow-up. The heighted risk was restricted to those diagnosed ≥20 years and those diagnosed ≥1995. Males (outcome n=12) did not have higher risk for neither incident nor prior anorexia nervosa.<br><br>There was also an increased odds for anorexia nervosa before the CeD diagnosis among women. aOR: 2.30 (1.48 to 3.58) (n=33/17,959 vs. 76/89,379), which was seen in both age groups but still restricted to those diagnosed ≥1995.

© 2026 Yao J et al. *JAMA Network Open*.

								However, only individuals with normal mucosa but positive CeD serology but not those with only intestinal inflammation had a higher risk for anorexia nervosa (in aHR). Both groups had higher odds for prior anorexia nervosa.
Butwicka A,[8]* 2017, Sweden;<br>Nationwide histopathology and specialized care register;<br>cases born from 1973 (start of psychiatric diagnoses records in the Swedish NPR) through 2008 and followed until 31 January 2010;<br>follow-up median (IQR): 9.6 years (5.3-15.6)	childhood-onset: CeD 10,903	Median (IQR): 3.0 years (1.3-8.9)	62.7	77 (0.7%)	Not reported	Included	Matching variables<br>1. age<br>2. sex<br>3. birth county<br>4. date of diagnosis<br><br>Additionally adjusted for:<br>5. maternal/paternal age at child's birth<br>6. maternal/paternal country of birth<br>7. level of education of higher-educated parent<br>8. gestational age<br>9. birth weight<br>10. birth cohort<br>11. Apgar score<br>(+only in the cohort study)<br>12. history of psychiatric disorders before recruitment	Children with CeD had a higher risk of developing any eating disorder before adulthood (aHR=1.4 [1.1 to 1.8]); this risk was robust among individuals free from any prior psychiatric disorder. On the other hand, CeD-free siblings did not have a higher risk for eating disorders. Children with CeD also had a higher odds for any prior eating disorder (aOR=2.8 [2.2 to 3.7])
Wotton CJ,[9]* 2016, UK;<br>National inpatient database across England;<br>1 April 1998 to 31 December 2011;<br>Follow-up duration not reported	8700[F], 651[M] with anorexia nervosa;<br><br>4783[F] and 330[M] with bulimia nervosa;<br><br>28,750[F] and 176,959[M] with any autoimmune disease (not specifically reported for CeD)	10-44 years;<br>in anorexia nervosa:<br>10-19 years: 49.3%[F]; 53.0%[M];<br>20-29 years: 28.8%[F]; 24.8%[M];<br>30-39 years: 16.0%[F]; 16.0%[M]<br>in bulimia nervosa:<br>10-19 years: 23.6%[F]; 22.1%[M];<br>20-29 years: 44.6%[F]; 44.0%[M];<br>30-39 years: 24.3%[F]; 28.4%[M]<br><br>in any autoimmune disease:<br>10-19 years: 9.8%[F]; 14.4%[M];	93.1 in anorexia nervosa;<br><br>93.5 in bulimia nervosa;<br><br>61.9 in any autoimmune disease	Anorexia nervosa: 16 female CeD;<br><br>Bulimia nervosa: 6 female CeD<br><br>CeD: 33 in female anorexia nervosa; 14 in female bulimia nervosa<br><br>Observations not explicitly reported for males	Not reported	Included	Standardization by the covariates below in calculating expected outcomes in each study group<br>1. age<br>2. sex<br>3. calendar year of the first recorded admission<br>4. region of residence<br>5. quintile of patients' Intex of Multiple Deprivation score for socioeconomic status	Females with CeD had a higher relative risk for later anorexia nervosa (aRR: 2.46 [1.40 to 4.03]) but not bulimia nervosa. The increased risk for anorexia nervsa was seen between one and four years after CeD diagnosis. There was also a positive association between CeD and anorexia nervosa in males (aRR: 10.3 [1.2 to 38.6], based on <5 outcomes)<br><br>Both anorexia nervosa and bulimia nervosa were associated with increased subsequent relative risk for CeD (aRR: 3.08 [2.11 to 4.34] and 2.41 [1.32 to 4.06], respectively). The increased risk were seen across investigated study period in females with anorexia nervosa, but limited to the first year of bulimia nervosa diagnosis. Relative risks in males not reported due to low outcome numbers.

© 2026 Yao J et al. *JAMA Network Open*.

		20-29 years: 23.4%[F]; 24.2%[M]; 30-39 years: 39.8%[F]; 37.4%[M]						

**Meta-analysis**			
**First author, publication year, updated to**	**Number of Studies Included**	**Heterogeneity I^2, %**	**Pooled Estimates (Mean (95%CI))**
Nikniaz Z,[10] 2021, Febuary 2021	23	For prevalence of bulimia nervosa in CeD: 95.44<br>For risk of anorexia nervosa in CeD: 0.00;<br>For risk of CeD in anorexia nervosa: 52.12;<br>I squared not explicitly reported for other estimates	In CeD, the pooled prevalence of any eating disorder was 8.9% [95% CI: 6.4% to 11.7%] (3.5% if any eating disorder was identified via diagnostic codes, 7.6% if CeD diagnosed via biopsy),<br>pooled prevalence of bulimia nervosa was 7.3% [95% CI: 0.2% to 21.6%].<br>The risk of anorexia nervosa in patients with CeD was significantly higher than in the healthy population (relative risk [RR]: 1.48 [95% CI: 1.32 to 1.64]).<br><br>In patients with anorexia nervosa and with any eating disorder, the pooled prevalence of CeD were 1.0% [95% CI: 0.2% to 2.3%] and 0.9% [95% CI: 0.6% to 1.3%], respectively. Moreover, the overall risk of subsequent CeD in patients with anorexia nervosa was significantly higher compared with healthy adults (RR: 2.35 [95% CI: 1.27 to 3.44]).

CeD: celiac disease; CI: confidence interval; [F]: female; (a)HR: (adjusted) hazard ratio; IQR: interquartile range; [M]: male; N/A: not applicable; NPR: National Patient Register; n.s.: not significant; (a)OR: (adjusted) odds ratio; RR: relative risk

* Included in the meta-analysis by Nikniaz et al[10]

© 2026 Yao J et al. *JAMA Network Open*.

eTable 2 SNOMED codes defining CeD [a]

Condition	Topographic codes	SNOMED codes [b]
Celiac disease (villus atrophy)	Duodenum: T64 (all);<br>Jejunum: T65, T65000 and T651 (excluding T652-9)	Celiac diagnosis: D6218x;<br>Marsh 3: M58, M5800, M58000, M58001, M58005, M58006, M58007
Mucosal healing	T64, T65, T65000, T651	Marsh 0: M00100, M00110,<br>Marsh 1-2: M40000, M41000, M42000, M43000, M47000, M47170

CeD: celiac disease; SNOMED: Systematized Nomenclature of Medicine;

[a] Diagnosis criterion: ≥1 SNOMED code for VA in the small intestine other than the ileum (PPV: 95% for biopsy samples between 1969 and 2008, and 99% for biopsy samples between 2009 and 2017)[11,12].

[b] In SNOMED codes, diagnostic codes that were listed under morphology in pathology registers starts with "D". For example D6218 was the diagnostic code for CeD. Meanwhile, codes that start with "M" (suggestive of inflammation but was not specific) in the table refer to all SNOMED codes starting with the respective code, which shall be accompanied by a topographic code.

The presence of persistent VA (Marsh 3) or mucosal healing (Marsh 0-2) was determined based on the follow-up biopsy result between six months and five years after the first biopsy that indicates Marsh 3 small intestinal VA.

© 2026 Yao J et al. *JAMA Network Open*.

eTable 3 Definitions of exclusion criteria

Exclusion	Data source	ICD-8 (1969-1986)	ICD-9 (1987-1996)	ICD-10 (1997-)
Disorders with eating behavior	NPR	305,50; 306,50	307B; 307F	F50
Chromosomal abnormalities	NPR	759,30; 759,50; 759;83; 310,5; 311,50; 312,50; 313,50; 314,50; 315,50	758	Q90-Q99

ICD: International Classification of Disease; NPR: National Patient Register.

© 2026 Yao J et al. *JAMA Network Open*.

eTable 4 Definitions of outcomes

Outcomes	Data source	ICD-10 (1997-)
Primary outcome		
Eating disorders	NPR	F500; F501; F502; F503; F509
Secondary outcomes		
Anorexia nervosa	NPR	F500 (typical anorexia nervosa); F501 (atypical anorexia nervosa)
Other eating disorders	NPR	F502 (bulimia nervosa); F503 (atypical bulimia nervosa); F509 (not otherwise specified)
Bulimia nervosa	NPR	F502; F503
Unspecified eating disorders	NPR	F509

ICD: International Classification of Disease; NPR: the National Patient Register

eTable 5 Definitions of comorbidities

Comorbidities	Data source	ICD-8 (1969-1986)	ICD-9 (1987-1996)	ICD-10 (1997-)
Autoimmune diseases				
Type 1 diabetes	NPR	250 (only if the first diagnosis ≤30 years [a])	250 (only if the first diagnosis ≤30 years [a])	E10
Inflammatory bowel disease	NPR	563; 563,0; 563,00; 563,1; 563,10; 563,9; 563,98; 563,99; 569,02; 569,04	555; 556	K50; K51; K523
Rheumatoid arthritis (individuals ≥16 years)	NPR	712,10; 712,20; 712,38; 712,39	714A; 714B; 714C; 714W; 719D	M05; M060; M062; M063; M068; M069; M123
Juvenile arthritis (individuals <16 years)	NPR	712,0	714D	M08
Any psychiatric disorders or suicide attempts	NPR	291; 295-299; 300-301; 303; 304; 307; 310-315; E950-E959	291; 295-298; 299A; 300; 301; 303; 304; 305A; 305X; 308-309; 311; 314; 317-319; E950-E959	F10-F45; F48; F60-F62; F69-F70; F84; F90; F91; X60-X84

ATC: anatomical therapeutic chemical; CeD: celiac disease; ERCP: Endoscopic retrograde cholangiopancreatography; ICD: International Classification of Disease; NPR: National Patient Register; PDR: Prescribed Drug Register;

[a] The algorithm for identifying the diagnosis of type 1 diabetes via ICD-8 and ICD-9 was proved to have a high positive predictive value (≥95%)[13].

© 2026 Yao J et al. *JAMA Network Open*.

eTable 6 Incident eating disorders in patients with CeD and their matched reference individuals

	No. of events (IR, per 100,000 person-years)		IR difference (95%CI), per 100,000 person-years	HR (95%CI)	
	Patients	Reference group		Model 1 [a]	Model 2 [b]
Primary outcome					
Any eating disorder	485 (117.4)	1578 (78.0)	39.5 (28.3 to 50.6)	1.50 (1.37 to 1.64)	1.39 (1.27 to 1.53)
Secondary outcomes					
Anorexia nervosa *	192 (46.3)	610 (30.0)	16.2 (9.3 to 23.2)	1.54 (1.34 to 1.78)	1.44 (1.24 to 1.67)
Other eating disorders	390 (94.3)	1258 (62.1)	32.2 (22.2 to 42.2)	1.51 (1.36 to 1.67)	1.39 (1.25 to 1.54)
Bulimia nervosa	68 (16.4)	239 (11.8)	4.6 (0.4 to 8.8)	1.41 (1.10 to 1.79)	1.23 (0.95 to 1.59)
Unspecified eating disorders	356 (86.0)	1106 (54.6)	31.5 (22.0 to 41.0)	1.56 (1.40 to 1.74)	1.45 (1.30 to 1.61)

CeD: celiac disease; CI: confidence interval; HR: hazard ratio; IR: incidence rate

[a] Model 1: conditioned on the matching variables (age, sex, county of residence, and calendar year of index date).

[b] Model 2: model 1 and further adjusted for country of birth, educational attainment, number of healthcare visits between two years and six months before the index date, and separately for the history of type 1 diabetes, inflammatory bowel disease, and juvenile idiopathic arthritis (<16 years)/rheumatoid arthritis (≥16 years).

*Anorexia nervosa cases were observed in 183 (IR: 69 per 100,000 person-years) females with CeD and 588 (IR: 46) female references, the aHR based on Model 2 was 1.42 (95%CI: 1.19 to 1.69). We present this female-specific result to facilitate the literature comparison

© 2026 Yao J et al. *JAMA Network Open*.

eTable 7 Cumulative incidence difference (95%CI) of incident eating disorders during follow-up in individuals with CeD, compared with their matched reference individuals

	**Difference in standardized cumulative incidence in percentages (95%CI), years since the index date [a]**			
	**1 year**	**5 years**	**10 years**	**20 years**
Primary outcome				
Any eating disorder	0.07 (0.04 to 0.10)	0.25 (0.17 to 0.32)	0.37 (0.24 to 0.50)	0.45 (0.22 to 0.69)
Secondary outcomes				
Anorexia nervosa	0.03 (0.01 to 0.05)	0.10 (0.05 to 0.15)	0.16 (0.07 to 0.25)	0.19 (0.03 to 0.35)
Other eating disorders	0.04 (0.02 to 0.06)	0.18 (0.12 to 0.25)	0.30 (0.18 to 0.41)	0.35 (0.15 to 0.56)

CeD: celiac disease; CI: confidence interval;

[a] Model 2: conditioned on the matching variables (age, sex, county of residence, and calendar year of index date) and further adjusted for country of birth, educational attainment, number of healthcare visits between two years and six months before the index date, and separately for the history of type 1 diabetes, inflammatory bowel disease, and juvenile idiopathic arthritis (<16 years)/rheumatoid arthritis (≥16 years).

© 2026 Yao J et al. *JAMA Network Open*.

eTable 8 Subgroup analyses of any incident eating disorder in patients with CeD and their matched reference individuals

Subgroup	No. of events (IR, per 100,000 person-years)		IR difference (95%CI), per 100,000 person-years	HR (95%CI)	
	Patients	Reference individuals		Model 1 [a]	Model 2 [b]
Age at index date, years					
6-<10	148 (180.4)	655 (164.1)	16.4 (-15.3 to 48.0)	1.09 (0.93 to 1.29)	1.04 (0.88 to 1.24)
10-<18	218 (303.5)	597 (167.1)	136.4 (93.9 to 178.8)	1.80 (1.57 to 2.06)	1.63 (1.41 to 1.88)
18-<30	91 (144.0)	245 (80.0)	64.0 (32.8 to 95.3)	1.82 (1.47 to 2.25)	1.70 (1.36 to 2.13)
≥30	28 (14.3)	81 (8.4)	5.9 (0.3 to 11.5)	1.62 (1.11 to 2.37)	1.46 (1.00 to 2.14)
Sex					
Female	452 (171.9)	1505 (117.1)	54.8 (37.9 to 71.7)	1.46 (1.33 to 1.60)	1.35 (1.23 to 1.49)
Male	33 (22.0)	73 (9.9)	12.1 (4.3 to 20.0)	2.30 (1.60 to 3.30)	2.30 (1.60 to 3.30)
Calendar period at index date					
2002-2012	336 (106.8)	1160 (75.2)	31.6 (19.4 to 43.9)	1.41 (1.27 to 1.57)	1.33 (1.19 to 1.49)
2013-2023	149 (151.4)	418 (86.9)	64.5 (38.8 to 90.2)	1.73 (1.47 to 2.04)	1.58 (1.33 to 1.87)
Educational attainment, years					
0-9	5 (11.4)	29 (13.3)	-1.9 (-13.0 to 9.1)	0.86 (0.33 to 2.24)	0.43 (0.07 to 2.53)
10-12	129 (83.6)	473 (63.1)	20.5 (5.0 to 36.0)	1.32 (1.11 to 1.57)	1.38 (1.10 to 1.73)
≥13	350 (164.3)	1075 (103.5)	60.7 (42.5 to 79.0)	1.59 (1.43 to 1.76)	1.38 (1.23 to 1.55)
Missing	≤2 (66.1)	≤2 (5.6)	60.4 (-69.5 to 190.3)	3.41 (0.27 to 43.3)	NA
Any history of type 1 diabetes, inflammatory bowel disease, or juvenile idiopathic arthritis/rheumatoid arthritis					
Yes	37 (110.2)	113 (66.7)	43.4 (5.9 to 81.0)	1.61 (1.16 to 2.23)	2.27 (0.33 to 15.77)
No	448 (118.1)	1465 (79.0)	39.1 (27.4 to 50.8)	1.49 (1.35 to 1.63)	1.42 (1.29 to 1.56)
Any history of psychiatric disorder or suicide attempt [c]					
Yes	57 (174.8)	56 (33.7)	141.1 (94.9 to 187.4)	5.12 (3.73 to 7.03)	4.80 (3.26 to 7.07)
No	428 (112.5)	1522 (81.9)	30.6 (19.2 to 42.0)	1.37 (1.24 to 1.50)	1.28 (1.16 to 1.41)

CeD: celiac disease; CI: confidence interval; HR: hazard ratio; IR: incidence rate

[a] Model 1: conditioned on the matching variables (age, sex, county of residence, and calendar year).

[b] Model 2: conditioned on covariates in model 1 and further adjusted for country of birth, educational attainment, number of healthcare visits between two years and six months before the index date, and separately for the history of type 1 diabetes, inflammatory bowel disease, and juvenile idiopathic arthritis (<16 years)/rheumatoid arthritis (≥16 years).

© 2026 Yao J et al. *JAMA Network Open*.

eTable 9 Sensitivity analyses of incident eating disorders in patients with CeD and their matched reference individuals

	No. of events (IR, per 100,000 person-years)		IR difference (95%CI), per 100,000 person-years	HR (95%CI) [a]
	Patients	Reference individuals		
Restricting to individuals with available educational attainment data				
Any eating disorder	484 (117.6)	1577 (78.5)	39.1 (28.0 to 50.3)	1.39 (1.27 to 1.53)
Anorexia nervosa	192 (46.4)	609 (30.2)	16.2 (9.2 to 23.2)	1.44 (1.24 to 1.67)
Other eating disorders	389 (94.4)	1258 (62.6)	31.8 (21.8 to 41.8)	1.38 (1.24 to 1.54)
Excluding first one year of follow-up				
Any eating disorder	434 (105.2)	1485 (73.5)	31.8 (21.2 to 42.4)	1.32 (1.19 to 1.45)
Anorexia nervosa	170 (41.0)	580 (28.6)	12.4 (5.8 to 19.0)	1.33 (1.13 to 1.56)
Other eating disorders	355 (85.9)	1185 (58.6)	27.4 (17.8 to 36.9)	1.33 (1.19 to 1.48)
Excluding first three years of follow-up				
Any eating disorder	352 (86.7)	1272 (63.9)	22.8 (13.0 to 32.5)	1.25 (1.12 to 1.39)
Anorexia nervosa	138 (33.8)	496 (24.8)	9.0 (2.9 to 15.0)	1.24 (1.04 to 1.48)
Other eating disorders	295 (72.5)	1019 (51.2)	21.4 (12.5 to 30.2)	1.30 (1.15 to 1.47)
≥2 diagnostic codes for eating disorders				
Any eating disorder	362 (87.5)	1134 (55.9)	31.5 (21.9 to 41.1)	1.44 (1.29 to 1.61)
Anorexia nervosa	156 (37.6)	473 (23.3)	14.3 (8.0 to 20.5)	1.47 (1.24 to 1.74)
Other eating disorders	268 (64.7)	822 (40.5)	24.1 (15.9 to 32.4)	1.45 (1.28 to 1.65)
Eating disorder diagnosis assigned by specialized psychiatric care				
Any eating disorder	423 (102.3)	1440 (71.1)	31.2 (20.8 to 41.6)	1.32 (1.19 to 1.46)
Anorexia nervosa	174 (41.9)	559 (27.5)	14.4 (7.8 to 21.0)	1.41 (1.21 to 1.66)
Other eating disorders	320 (77.3)	1115 (55.0)	22.3 (13.2 to 31.3)	1.28 (1.14 to 1.44)
Additionally adjusted for any history of psychiatric disorders or suicide attempt				
Any eating disorder	485 (117.4)	1578 (78.0)	39.5 (28.3 to 50.6)	1.40 (1.27 to 1.54)
Anorexia nervosa	192 (46.3)	610 (30.0)	16.2 (9.3 to 23.2)	1.45 (1.24 to 1.68)
Other eating disorders	390 (94.3)	1258 (62.1)	32.2 (22.2 to 42.2)	1.40 (1.26 to 1.55)
Additionally adjusted for any history of psychiatric disorders or suicide attempt among any of parents or full siblings				
Any eating disorder	485 (117.4)	1578 (78.0)	39.5 (28.3 to 50.6)	1.40 (1.27 to 1.54)
Anorexia nervosa	192 (46.3)	610 (30.0)	16.2 (9.3 to 23.2)	1.44 (1.24 to 1.67)
Other eating disorders	390 (94.3)	1258 (62.1)	32.2 (22.2 to 42.2)	1.40 (1.26 to 1.55)
Using attained age as underlying time scale				
Any eating disorder	485 (117.4)	1578 (78.0)	39.5 (28.3 to 50.6)	1.39 (1.27 to 1.53)
Anorexia nervosa	192 (46.3)	610 (30.0)	16.2 (9.3 to 23.2)	1.42 (1.22 to 1.65)
Other eating disorders	390 (94.3)	1258 (62.1)	32.2 (22.2 to 42.2)	1.39 (1.25 to 1.55)

CeD: celiac disease; CI: confidence interval; HR: hazard ratio; IR: incidence rate

[a] conditioned on matching variables (age, sex, county of residence, and calendar year) and further adjusted for country of birth, educational attainment, number of healthcare visits between two years and six months before the index date, and separately for the history of type 1 diabetes, inflammatory bowel disease, and juvenile idiopathic arthritis (<16 years)/rheumatoid arthritis (≥16 years).

© 2026 Yao J et al. *JAMA Network Open*.

eTable 10 Sensitivity analyses on eating disorders before the diagnosis of CeD, among individuals diagnosed ≥6 years of age

	With/without eating disorders		OR (95%CI) [a]
	Patients	Reference individuals	
**Any prior eating disorder**			
Any eating disorder	256/33,467	844/165,261	1.30 (1.12 to 1.52)
Anorexia nervosa	92/33,631	288/165,817	1.32 (1.03 to 1.70)
Other eating disorders	201/33,522	682/165,423	1.26 (1.06 to 1.49)
**Eating disorders diagnosed >1 year before the index date**			
Any eating disorder	186/33,537	763/165,342	1.04 (0.87 to 1.23)
Anorexia nervosa	66/33,657	259/165,846	1.04 (0.78 to 1.39)
Other eating disorders	145/33,578	618/165,487	1.00 (0.82 to 1.22)

CeD: celiac disease; CI: confidence interval; OR: odds ratio

[a] conditioned on matching variables (age, sex, county of residence, and calendar year) and further adjusted for country of birth, educational attainment, number of healthcare visits between two years and six months before the index date, and separately for the history of type 1 diabetes, inflammatory bowel disease, and juvenile idiopathic arthritis (<16 years)/rheumatoid arthritis (≥16 years).

© 2026 Yao J et al. *JAMA Network Open*.

eTable 11 Characteristics of CeD patients who had a follow-up biopsy between six months and five years after diagnosis, n (%)

	Persistent VA	Mucosal healing
N	2344	6798
Age at index date, years [a]		
Mean ± SD	49.0 ± 21.3	36.4 ± 21.1
Median (IQR)	50.7 (32.1 - 67.1)	33.8 (20.0 - 52.6)
6-<10	80 (3.4%)	694 (10.2%)
10-<18	111 (4.7%)	646 (9.5%)
18-<30	308 (13.1%)	1526 (22.4%)
≥30	1845 (78.7%)	3932 (57.8%)
Female	1389 (59.3%)	4507 (66.3%)
Born in Nordic countries [b]	2153 (91.9%)	6367 (93.7%)
Calendar period at index date		
2002-2012	1288 (54.9%)	3731 (54.9%)
2013-2023	1056 (45.1%)	3067 (45.1%)
Educational attainment, years		
0-9	449 (19.2%)	666 (9.8%)
10-12	974 (41.6%)	2654 (39.0%)
≥13	897 (38.3%)	3427 (50.4%)
Missing	24 (1.0%)	51 (0.8%)
Number of healthcare visits [c]		
0	246 (10.5%)	810 (11.9%)
1	401 (17.1%)	1205 (17.7%)
2-3	719 (30.7%)	2112 (31.1%)
≥4	978 (41.7%)	2671 (39.3%)
History of any following autoimmune diseases [d]		
Type 1 diabetes	79 (3.4%)	266 (3.9%)
Inflammatory bowel disease	96 (4.1%)	367 (5.4%)
Juvenile arthritis or rheumatoid arthritis	30 (1.3%)	71 (1.0%)
History of psychiatric disorder or suicide attempts [d]	287 (12.2%)	866 (12.7%)
Follow-up time, years		
Mean ± SD	10.3 ± 5.69	10.9 ± 5.65
Median (IQR)	10.1 (5.3 - 15.2)	11.0 (6.0 - 15.8)

© 2026 Yao J et al. *JAMA Network Open*.

	Persistent VA	Mucosal healing
0-0.9	61 (2.6%)	124 (1.8%)
1-4.9	478 (20.4%)	1260 (18.5%)
5-9.9	611 (26.1%)	1676 (24.7%)
10-14.9	584 (24.9%)	1710 (25.2%)
≥15	610 (26.0%)	2028 (29.8%)
Among individuals diagnosed with any eating disorder during follow-up		
N (% of all included individuals)	10 (0.4%)	76 (1.1%)
Age at eating disorder diagnosis, years		
Mean ± SD	29.6 ± 13.3	22.8 ± 9.5
Median (IQR)	26.5 (19.8 - 38.8)	20.0 (16.1 - 28.7)
6-<18 (% of individuals diagnosed with any eating disorder, same as below)	1 (10.0%)	24 (31.6%)
18-<30	5 (50.0%)	35 (46.1%)
≥30	4 (40.0%)	17 (22.4%)

VA: villus atrophy; IQR: interquartile range: SD: standard deviation.
[a] Date of follow-up biopsy.
[b] Nordic countries: Sweden, Denmark, Finland, Norway, and Iceland.
[c] Between two years and six months before the index date.
[d] Codes for disease histories are listed in **eTable 5**.

© 2026 Yao J et al. *JAMA Network Open*.

eTable 12 Incident eating disorders in CeD patients who had persistent VA compared with those with mucosal healing

	No. of events (IR, per 100,000 person-years)		IR difference (95%CI), per 100,000 person-years	HR (95%CI)	
	Persistent VA	Mucosal healing		Model 1 [a]	Model 2 [b]
Primary outcome					
Any eating disorder	10 (41.3)	76 (102.5)	-61.2 (-95.6 to -26.8)	0.64 (0.33 to 1.26)	0.65 (0.33 to 1.28)
Secondary outcomes					
Anorexia nervosa	≤2 (8.2)	27 (36.2)	-28.0 (-45.8 to -10.2)	0.38 (0.09 to 1.64)	0.39 (0.09 to 1.66)
Other eating disorders	10 (41.3)	66 (88.8)	-47.6 (-80.9 to -14.2)	0.73 (0.37 to 1.44)	0.73 (0.37 to 1.45)
Bulimia nervosa	≤2 (4.1)	13 (17.4)	-13.3 (-25.7 to -0.9)	0.43 (0.05 to 3.41)	0.38 (0.05 to 3.11)
Unspecified eating disorders	9 (37.1)	59 (79.4)	-42.2 (-73.8 to -10.7)	0.69 (0.34 to 1.43)	0.71 (0.34 to 1.46)

CeD: celiac disease; CI: confidence interval; HR: hazard ratio; IR: incidence rate; VA: villus atrophy

[a] Model 1: conditioned on the matching variables (age, sex, county of residence, and calendar year of index date).

[b] Model 2: model 1 and further adjusted for country of birth, educational attainment, number of healthcare visits between two years and six months before the index date, and separately for the history of type 1 diabetes, inflammatory bowel disease, and juvenile idiopathic arthritis (<16 years)/rheumatoid arthritis (≥16 years), as well as duration between biopsy indicating VA and the follow-up biopsy.

© 2026 Yao J et al. *JAMA Network Open*.

eTable 13 Incident eating disorders in bFDRs of patients with CeD and their matched reference individuals

	No. of events (IR, per 100,000 person-years)		IR difference (95%CI), per 100,000 person-years	HR (95%CI)	
	Parents of				
	Patients	Reference individuals		Model 1 [a]	Model 2 [b]
Primary outcome					
Any eating disorder	51 (8.5)	260 (9.1)	-0.6 (-3.2 to 2.0)	0.97 (0.72 to 1.31)	0.82 (0.61 to 1.12)
Secondary outcomes					
Anorexia nervosa	4 (0.7)	61 (2.1)	-1.5 (-2.3 to -0.6)	0.33 (0.12 to 0.89)	0.27 (0.10 to 0.77)
Other eating disorders	48 (8.0)	229 (8.0)	-0.03 (-2.5 to 2.5)	1.04 (0.76 to 1.42)	0.87 (0.64 to 1.19)
Bulimia nervosa	13 (2.2)	67 (2.4)	-0.2 (-1.5 to 1.1)	0.97 (0.53 to 1.76)	0.80 (0.44 to 1.44)
Unspecified eating disorders	36 (6.0)	191 (6.7)	-0.7 (-2.9 to 1.5)	0.94 (0.65 to 1.34)	0.79 (0.55 to 1.13)
	**No. of events (IR, per 100,000 person-years)**		**IR difference (95%CI), per 100,000 person-years**	**HR (95%CI)**	
	**Full siblings of**				
	**Patients**	**Reference individuals**		**Model 1 [a]**	**Model 2 [b]**
Primary outcome					
Any eating disorder	389 (70.7)	1570 (61.1)	9.6 (1.9 to 17.2)	1.19 (1.07 to 1.34)	1.11 (0.99 to 1.24)
Secondary outcomes					
Anorexia nervosa	162 (29.4)	621 (24.1)	5.3 (0.4 to 10.2)	1.26 (1.06 to 1.50)	1.20 (1.00 to 1.43)
Other eating disorders	307 (55.7)	1254 (48.8)	7.0 (0.2 to 13.8)	1.18 (1.04 to 1.34)	1.08 (0.95 to 1.23)
Bulimia nervosa	56 (10.1)	215 (8.3)	1.8 (-1.1 to 4.7)	1.24 (0.92 to 1.67)	1.15 (0.85 to 1.54)
Unspecified eating disorders	273 (49.6)	1132 (44.0)	5.5 (-0.9 ro 12.0)	1.16 (1.01 to 1.33)	1.06 (0.93 to 1.22)
	**No. of events (IR, per 100,000 person-years)**		**IR difference (95%CI), per 100,000 person-years**	**HR (95%CI)**	
	**Offspring of**				
	**Patients**	**Reference individuals**		**Model 1 [a]**	**Model 2 [b]**
Primary outcome					
Any eating disorder	277 (66.7)	1167 (59.0)	7.6 (-0.9 to 16.2)	1.12 (0.98 to 1.27)	1.05 (0.92 to 1.20)
Secondary outcomes					
Anorexia nervosa	101 (24.2)	471 (23.8)	0.5 (-4.7 to 5.7)	1.01 (0.81 to 1.26)	0.94 (0.75 to 1.17)
Other eating disorders	232 (55.8)	925 (46.7)	9.1 (1.3 to 16.8)	1.18 (1.02 to 1.36)	1.10 (0.95 to 1.28)
Bulimia nervosa	46 (11.0)	174 (8.8)	2.3 (-1.2 to 5.7)	1.23 (0.89 to 1.70)	1.16 (0.83 to 1.60)
Unspecified eating disorders	208 (50.0)	814 (41.1)	8.8 (1.5 to 16.2)	1.20 (1.03 to 1.40)	1.13 (0.97 to 1.32)

CeD: celiac disease; CI: confidence interval; bFDR: biological first-degree relative; HR: hazard ratio; IR: incidence rate

[a] Model 1: conditioned on the matching variables (age, sex, county of residence, and calendar year of index date) and a family identifier.

[b] Model 2: model 1 and further adjusted for country of birth, educational attainment, number of healthcare visits between two years and six months before the index date, the history of type 1 diabetes, inflammatory bowel disease, and juvenile idiopathic arthritis (<16 years)/rheumatoid arthritis (≥16 years). Additionally, we adjusted for their index family members' age, sex, number of healthcare visits between two years and six months before the index date, and conditioned on a family identifier.

eTable 14 Cumulative incidence difference (95%CI) of incident eating disorders during follow-up in bFDRs of patients with CeD and their matched reference individuals

© 2026 Yao J et al. *JAMA Network Open*.

	Standardized cumulative incidence difference in percentages (95%CI) among parents of patients with CeD vs. of reference individuals, years since the index date [a]			
	1 year	5 years	10 years	20 years
Primary outcome				
Any eating disorder	-0.002 (-0.008 to 0.005)	-0.010 (-0.028 to 0.009)	-0.019 (-0.044 to 0.006)	-0.021 (-0.060 to 0.019)
Secondary outcomes				
Anorexia nervosa	0.000 (-0.003 to 0.002)	-0.008 (-0.015 to -0.002)	-0.014 (-0.023 to -0.006)	-0.024 (-0.036 to -0.011)
Other eating disorders	0.000 (-0.007 to 0.006)	-0.006 (-0.02 to 0.01)	-0.016 (-0.039 to 0.008)	-0.010 (-0.049 to 0.028)

	Standardized cumulative incidence difference in percentages (95%CI) among full siblings of patients with CeD vs. of reference individuals, years since the index date [a]			
	1 year	5 years	10 years	20 years
Primary outcome				
Any eating disorder	0.013 (-0.005 to 0.032)	0.047 (-0.003 to 0.098)	0.082 (-0.011 to 0.176)	0.104 (-0.066 to 0.274)
Secondary outcomes				
Anorexia nervosa	0.012 (0.000 to 0.256)	0.047 (0.012 to 0.081)	0.077 (0.009 to 0.146)	0.030 (-0.089 to 0.149)
Other eating disorders	0.038 (-0.010 to 0.018)	0.017 (-0.027 to 0.061)	0.048 (-0.033 to 0.129)	0.075 (-0.073 to 0.222)

	Standardized cumulative incidence difference in percentages (95%CI) among offspring of patients with CeD vs. of reference individuals, years since the index date [a]			
	1 year	5 years	10 years	20 years
Primary outcome				
Any eating disorder	0.014 (-0.005 to 0.034)	0.009 (-0.041 to 0.058)	-0.003 (-0.114 to 0.108)	0.140 (-0.082 to 0.362)
Secondary outcomes				
Anorexia nervosa	0.009 (-0.004 to 0.021)	0.008 (-0.025 to 0.040)	-0.057 (-0.138 to 0.024)	0.002 (-0.173 to 0.176)
Other eating disorders	0.004 (-0.011 to 0.020)	-0.005 (-0.048 to 0.038)	0.031 (-0.066 to 0.127)	0.175 (-0.017 to 0.366)

CeD: celiac disease; CI: confidence interval; bFDR: biological first-degree relative.

[a] Model 2: conditioned on the matching variables (age, sex, county of residence, and calendar year of index date) and further adjusted for country of birth, educational attainment, number of healthcare visits between two years and six months before the index date, and separately for the history of type 1 diabetes, inflammatory bowel disease, and juvenile idiopathic arthritis (<16 years)/rheumatoid arthritis (≥16 years). Additionally, we adjusted for their index family members' age, sex, number of healthcare visits between two years and six months before the index date, and conditioned on a family identifier.

© 2026 Yao J et al. *JAMA Network Open*.

eTable 15 Subgroup analyses of any incident eating disorder in bFDRs of patients with CeD and their matched reference individuals

Subgroup	No. of events (IR, per 100,000 person-years)		IR difference (95%CI), per 100,000 person-years	HR (95%CI)	
	Patients	Parents of Reference individuals		Model 1 [a]	Model 2 [b]
Age at index date, years					
6-<10	0 (0.0)	0 (0.0)	N/A	N/A	N/A
10-<18	0 (0.0)	0 (0.0)	N/A	N/A	N/A
18-<30	8 (36.3)	51 (45.5)	-9.2 (-37.3 to 18.8)	0.83 (0.39 to 1.73)	0.62 (0.29 to 1.34)
≥30	43 (7.4)	209 (7.6)	-0.2 (-2.6 to 2.3)	1.00 (0.72 to 1.40)	0.89 (0.64 to 1.24)
Sex					
Female	47 (15.0)	240 (16.0)	-1.0 (-5.8 to 3.7)	0.98 (0.71 to 1.34)	0.83 (0.60 to 1.13)
Male	4 (1.4)	20 (1.5)	-0.1 (-1.6 to 1.4)	0.94 (0.32 to 2.77)	0.80 (0.29 to 2.24)
Calendar period at index date					
2002-2012	43 (9.2)	223 (10.0)	-0.8 (-3.9 to 2.3)	0.95 (0.69 to 1.33)	0.81 (0.58 to 1.12)
2013-2023	8 (6.0)	37 (6.0)	0.1 (-4.5 to 4.7)	1.09 (0.51 to 2.33)	0.95 (0.43 to 2.08)
Educational attainment, years					
0-9	9 (8.8)	50 (9.3)	-0.6 (-6.9 to 5.7)	1.15 (0.56 to 2.39)	0.81 (0.39 to 1.68)
10-12	23 (8.2)	123 (9.2)	-1.0 (-4.7 to 2.7)	0.91 (0.58 to 1.43)	0.82 (0.52 to 1.30)
≥13	19 (9.0)	87 (9.3)	-0.2 (-4.7 to 4.3)	1.00 (0.61 to 1.66)	0.90 (0.54 to 1.49)
Missing	0 (0.0)	0 (0.0)	N/A	N/A	N/A
Any history of type 1 diabetes, inflammatory bowel disease, or juvenile idiopathic arthritis/rheumatoid arthritis					
Yes	5 (20.3)	13 (16.7)	3.6 (-16.4 to 23.6)	1.44 (0.52 to 4.00)	1.36 (0.48 to 3.81)
No	46 (8.0)	247 (8.9)	-0.9 (-3.5 to 1.7)	0.93 (0.68 to 1.28)	0.81 (0.59 to 1.11)
Any history of psychiatric disorder or suicide attempt					
Yes	21 (40.8)	106 (41.2)	-0.4 (-19.5 to 18.7)	1.06 (0.66 to 1.70)	0.89 (0.55 to 1.45)
No	30 (5.5)	154 (5.9)	-0.5 (-2.6 to 1.7)	0.96 (0.65 to 1.42)	0.84 (0.57 to 1.24)
Index family member (offspring)'s age of CeD diagnosis/matching, years					
<18	46 (13.6)	226 (13.8)	-0.1 (-0.4 to 4.2)	1.02 (0.74 to 1.40)	0.86 (0.62 to 1.18)
18-<30	4 (3.3)	23 (4.1)	-0.8 (-4.4 to 2.8)	0.86 (0.30 to 2.51)	0.72 (0.25 to 2.10)
≥30	≤2 (0.7)	11 (1.7)	-0.1 (-2.7 to 0.7)	0.41 (0.05 to 3.30)	0.34 (0.11 to 1.02)
Index family member (offspring)'s sex					
Female	30 (7.6)	174 (9.3)	-1.7 (-4.7 to 1.3)	0.85 (0.58 to 1.26)	0.71 (0.48 to 1.06)
Male	21 (10.3)	86 (8.8)	1.5 (-3.3 to 6.3)	1.22 (0.75 to 1.96)	1.10 (0.68 to 1.77)

© 2026 Yao J et al. *JAMA Network Open*.

Subgroup	No. of events (IR, per 100,000 person-years) Full siblings of Patients	Reference individuals	IR difference (95%CI), per 100,000 person-years	HR (95%CI) Model 1 [a]	Model 2 [b]
Age at index date, years					
6-<10	140 (140.7)	611 (126.6)	14.0 (-11.3 to 39.4)	1.09 (0.91 to 1.32)	1.01 (0.84 to 1.22)
10-<18	175 (183.2)	633 (133.7)	49.5 (20.4 to 78.6)	1.38 (1.17 to 1.64)	1.27 (1.07 to 1.51)
18-<30	51 (52.7)	247 (53.2)	-0.5 (-16.4 to 15.4)	1.01 (0.74 to 1.36)	0.95 (0.70 to 1.28)
≥30	23 (8.9)	79 (6.9)	2.0 (-1.9 to 6.0)	1.29 (0.81 to 2.06)	1.25 (0.78 to 2.01)
Sex					
Female	358 (132.2)	1443 (114.6)	17.6 (2.7 to 32.5)	1.20 (1.06 to 1.34)	1.11 (0.99 to 1.25)
Male	31 (11.1)	127 (9.7)	1.4 (-2.9 to 5.7)	1.19 (0.80 to 1.76)	1.06 (0.70 to 1.61)
Calendar period at index date					
2002-2012	273 (65.2)	1131 (57.9)	7.3 (-1.2 to 15.7)	1.16 (1.01 to 1.32)	1.08 (0.95 to 1.24)
2013-2023	116 (88.1)	439 (71.2)	17.0 (-0.4 to 34.3)	1.29 (1.05 to 1.59)	1.17 (0.95 to 1.45)
Educational attainment, years					
0-9	10 (17.2)	39 (13.5)	3.7 (-7.8 to 15.1)	1.67 (0.85 to 3.32)	1.38 (0.71 to 2.68)
10-12	108 (49.5)	407 (39.1)	10.4 (0.3 to 20.5)	1.37 (1.11 to 1.70)	1.28 (1.03 to 1.59)
≥13	271 (99.6)	1123 (91.3)	8.3 (-4.7 to 21.3)	1.11 (0.97 to 1.27)	1.04 (0.91 to 1.19)
Missing	0 (0.0)	≤2 (11.9)	-11.9 (-35.3 to 11.5)	N/A	N/A
Any history of type 1 diabetes, inflammatory bowel disease, or juvenile idiopathic arthritis/rheumatoid arthritis					
Yes	10 (61.0)	17 (35.6)	25.5 (-16.0 to 66.9)	1.35 (0.59 to 3.08)	1.35 (0.50 to 3.62)
No	379 (71.0)	1553 (61.6)	9.4 (1.6 to 17.2)	1.19 (1.06 to 1.33)	1.11 (0.99 to 1.24)
Any history of psychiatric disorder or suicide attempt					
Yes	46 (124.0)	141 (83.4)	40.7 (2.3 to 79.1)	1.58 (1.13 to 2.20)	1.52 (1.08 to 2.13)
No	343 (66.8)	1429 (59.5)	7.3 (-0.4 to 15.0)	1.16 (1.03 to 1.30)	1.08 (0.96 to 1.22)
Index family member (sibling)'s age of CeD diagnosis/matching, years					
<18	300 (149.4)	1220 (121.5)	28.0 (9.7 to 46.2)	1.21 (1.07 to 1.38)	1.12 (0.98 to 1.28)
18-<30	63 (67.6)	254 (60.0)	7.6 (-10.6 to 25.9)	1.17 (0.88 to 1.54)	1.07 (0.81 to 1.42)
≥30	26 (10.1)	96 (8.4)	1.7 (-2.5 to 6.0)	1.20 (0.78 to 1.85)	1.18 (0.76 to 1.82)
Index family member (sibling)'s sex					
Female	253 (72.7)	1012 (62.0)	10.7 (1.0 to 20.5)	1.21 (1.05 to 1.40)	1.13 (0.98 to 1.30)
Male	136 (67.2)	558 (59.5)	7.7 (-4.7 to 20.0)	1.16 (0.96 to 1.40)	1.08 (0.89 to 1.31)

Subgroup	No. of events (IR, per 100,000 person-years) Offspring of	IR difference (95%CI), per 100,000 person-years	HR (95%CI) Model 1 [a]

© 2026 Yao J et al. *JAMA Network Open*.

	Patients	Reference individuals			Model 2 [b]
Age at index date, years					
6-<10	126 (138.7)	476 (112.7)	26.0 (-0.3 to 52.2)	1.20 (0.99 to 1.47)	1.14 (0.93 to 1.39)
10-<18	82 (128.2)	422 (139.6)	-11.4 (-42.2 to 19.4)	0.91 (0.72 to 1.15)	1.27 (0.93 to 1.73)
18-<30	51 (62.1)	203 (50.1)	12.1 (-6.3 to 30.4)	1.27 (0.93 to 1.73)	1.24 (0.91 to 1.70)
≥30	18 (10.1)	66 (7.8)	2.3 (-2.7 to 7.3)	1.30 (0.77 to 2.20)	1.21 (0.71 to 2.05)
Sex					
Female	246 (121.9)	1075 (111.8)	10.1 (-6.6 to 26.7)	1.07 (0.93 to 1.23)	1.01 (0.87 to 1.16)
Male	31 (14.5)	92 (9.1)	5.5 (0.02 to 10.9)	1.62 (1.08 to 2.43)	1.57 (1.05 to 2.34)
Calendar period at index date					
2002-2012	186 (60.4)	768 (52.9)	7.5 (-2.0 to 16.9)	1.10 (0.94 to 1.30)	1.05 (0.89 to 1.23)
2013-2023	91 (84.6)	399 (75.9)	8.7 (-10.2 to 27.6)	1.14 (0.90 to 1.43)	1.05 (0.83 to 1.32)
Educational attainment, years					
0-9	23 (65.2)	140 (74.0)	-8.8 (-38.2 to 20.5)	0.95 (0.61 to 1.49)	0.89 (0.57 to 1.40)
10-12	75 (45.9)	323 (40.4)	5.5 (-5.8 to 16.7)	1.10 (0.86 to 1.43)	1.07 (0.82 to 1.38)
≥13	155 (83.1)	560 (68.2)	15.0 (0.7 to 29.2)	1.12 (0.94 to 1.33)	1.08 (0.90 to 1.29)
Missing	24 (78.8)	144 (85.4)	-6.6 (-41.1 to 27.9)	0.99 (0.64 to 1.52)	0.94 (0.60 to 1.46)
Any history of type 1 diabetes, inflammatory bowel disease, or juvenile idiopathic arthritis/rheumatoid arthritis					
Yes	3 (27.9)	17 (53.7)	-25.8 (-66.4 to 14.8)	0.41 (0.11 to 1.51)	0.11 (0.03 to 0.48)
No	274 (67.7)	1150 (59.1)	8.6 (-0.1 to 17.3)	1.13 (0.99 to 1.29)	1.06 (0.93 to 1.22)
Any history of psychiatric disorder or suicide attempt					
Yes	27 (101.7)	110 (86.8)	14.9 (-26.8 to 56.5)	1.18 (0.77 to 1.82)	1.20 (0.77 to 1.85)
No	250 (64.3)	1057 (57.1)	7.2 (-1.5 to 15.8)	1.11 (0.96 to 1.27)	1.04 (0.91 to 1.20)
Index family member (parents)’s age of CeD diagnosis/matching, years					
<18	0 (0.0)	≤2 (10.8)	-10.8 (-31.9 to 10.3)	N/A	N/A
18-<30	29 (136.9)	88 (81.6)	55.4 (2.7 to 108.0)	1.68 (1.12 to 2.52)	1.75 (1.16 to 2.65)
≥30	248 (63.1)	1078 (57.9)	5.2 (-3.4 to 13.8)	1.07 (0.93 to 1.23)	1.01 (0.87 to 1.16)
Index family member (parent)'s sex					
Female	186 (71.4)	755 (61.4)	10.1 (-1.1 to 21.2)	1.17 (0.99 to 1.37)	1.10 (0.93 to 1.29)
Male	91 (58.6)	412 (55.1)	3.5 (-9.7 to 16.7)	1.02 (0.81 to 1.29)	0.95 (0.75 to 1.20)

CeD: celiac disease; CI: confidence interval; bFDR: biological first-degree relative; HR: hazard ratio; IR: incidence rate

[a] Model 1: conditioned on the matching variables (age, sex, county of residence, and calendar year) and a family identifier.

[b] Model 2: conditioned on covariates in model 1 and further adjusted for country of birth, educational attainment, number of healthcare visits between two years and six months before the index date, and separately for the history of type 1 diabetes, inflammatory bowel disease, and juvenile idiopathic arthritis (<16 years)/rheumatoid arthritis (≥16 years). Additionally, we adjusted for their index family members’ age, sex, number of healthcare visits between two years and six months before the index date, and conditioned on a family identifier.

© 2026 Yao J et al. *JAMA Network Open*.

eTable 16 Sensitivity analyses of incident eating disorders in bFDRs of patients with CeD and their matched reference individuals

	No. of events (IR, per 100,000 person-years)			
	Parents of			
	Patients	Reference individuals	IR difference (95%CI), per 100,000 person-years	HR (95%CI) [a]
Restricting to individuals without CeD				
Any eating disorder	50 (8.5)	260 (9.1)	-0.6 (-3.2 to 2.0)	0.83 (0.61 to 1.13)
Anorexia nervosa	4 (0.7)	61 (2.1)	-1.5 (-2.3 to -0.6)	0.28 (0.10 to 0.79)
Other eating disorders	47 (8.0)	229 (8.0)	-0.02 (-2.5 to 2.5)	0.88 (0.64 to 1.21)
Restricting to individuals with available educational attainment data				
Any eating disorder	51 (8.6)	260 (9.2)	-0.7 (-3.3 to 1.9)	0.82 (0.61 to 1.12)
Anorexia nervosa	4 (0.7)	61 (2.2)	-1.5 (-2.3 to -0.6)	0.27 (0.10 to 0.77)
Other eating disorders	48 (8.1)	229 (8.1)	-0.07 (-2.6 to 2.4)	0.87 (0.64 to 1.19)
Excluding first one year of follow-up				
Any eating disorder	45 (7.5)	237 (8.3)	-0.8 (-3.3 to 1.6)	0.81 (0.58 to 1.11)
Anorexia nervosa	3 (0.5)	57 (2.0)	-1.5 (-2.3 to -0.7)	0.22 (0.06 to 0.73)
Other eating disorders	42 (7.0)	209 (7.3)	-0.3 (-2.7 to 2.0)	0.84 (0.60 to 1.17)
Excluding first three years of follow-up				
Any eating disorder	35 (5.9)	173 (6.2)	-0.2 (-2.4 to 1.9)	0.85 (0.59 to 1.23)
Anorexia nervosa	≤2 (0.3)	40 (1.4)	-1.1 (-1.7 to -0.4)	0.20 (0.05 to 0.87)
Other eating disorders	33 (5.6)	157 (5.6)	-0.01 (-2.1 to 2.1)	0.87 (0.60 to 1.27)
≥2 diagnostic codes for eating disorders				
Any eating disorder	29 (4.8)	166 (5.8)	-1.0 (-3.0 to 1.0)	0.73 (0.49 to 1.09)
Anorexia nervosa	3 (0.5)	40 (1.4)	-0.9 (-1.6 to -0.2)	0.33 (0.10 to 1.10)
Other eating disorders	26 (4.3)	142 (5.0)	-0.6 (-2.5 to 1.2)	0.75 (0.49 to 1.15)
Eating disorder diagnosis assigned by specialized psychiatric care				
Any eating disorder	43 (7.2)	226 (7.9)	-0.8 (-3.1 to 1.6)	0.80 (0.58 to 1.12)
Anorexia nervosa	3 (0.5)	48 (1.7)	-1.2 (-1.9 to -0.4)	0.26 (0.08 to 0.87)
Other eating disorders	40 (6.7)	204 (7.2)	-0.5 (-2.8 to 1.8)	0.82 (0.58 to 1.15)
Using attained age as underlying time scale				
Any eating disorder	51 (8.5)	260 (9.1)	-0.6 (-3.2 to 2.0)	0.83 (0.61 to 1.12)
Anorexia nervosa	4 (0.7)	61 (2.1)	-1.5 (-2.3 to -0.6)	0.27 (0.10 to 0.77)
Other eating disorders	48 (8.0)	229 (8.0)	-0.03 (-2.5 to 2.5)	0.88 (0.64 to 1.20)

	No. of events (IR, per 100,000 person-years)			
	Full siblings of		IR difference (95%CI), per 100,000 person-years	HR (95%CI) [a]

© 2026 Yao J et al. *JAMA Network Open*.

	Patients	Reference individuals		
Restricting to individuals without CeD				
Any eating disorder	350 (67.1)	1560 (60.9)	6.2 (-1.5 to 13.8)	1.12 (0.99 to 1.26)
Anorexia nervosa	145 (27.7)	617 (24.0)	3.7 (-1.2 to 8.6)	1.20 (1.00 to 1.44)
Other eating disorders	280 (53.6)	1246 (48.6)	5.0 (-1.8 to 11.9)	1.11 (0.97 to 1.26)
Restricting to individuals with available educational attainment data				
Any eating disorder	389 (70.9)	1569 (61.3)	9.6 (2.0 to 17.3)	1.10 (0.96 to 1.25)
Anorexia nervosa	162 (29.4)	620 (24.2)	5.3 (0.4 to 10.2)	1.23 (0.99 to 1.52)
Other eating disorders	307 (55.9)	1253 (48.9)	7.0 (0.2 to 13.8)	1.05 (0.91 to 1.22)
Excluding first one year of follow-up				
Any eating disorder	363 (66.0)	1478 (57.6)	8.5 (1.0 to 15.9)	1.10 (0.98 to 1.24)
Anorexia nervosa	152 (27.6)	590 (22.9)	4.7 (-0.1 to 9.4)	1.18 (0.98 to 1.41)
Other eating disorders	289 (52.5)	1183 (46.1)	6.5 (-0.1 to 13.1)	1.09 (0.95 to 1.24)
Excluding first three years of follow-up				
Any eating disorder	297 (54.8)	1232 (48.7)	6.1 (-0.7 to 12.9)	1.09 (0.95 to 1.24)
Anorexia nervosa	117 (21.5)	498 (19.6)	1.9 (-2.4 to 6.2)	1.08 (0.88 to 1.33)
Other eating disorders	247 (45.5)	994 (39.3)	6.2 (-0.07 to 12.4)	1.11 (0.96 to 1.28)
≥2 diagnostic codes for eating disorders				
Any eating disorder	297 (53.9)	1139 (44.3)	9.6 (3.0 to 16.3)	1.16 (1.02 to 1.32)
Anorexia nervosa	134 (24.3)	484 (18.8)	5.5 (1.1 to 9.9)	1.27 (1.04 to 1.54)
Other eating disorders	219 (39.7)	811 (31.5)	8.2 (2.5 to 13.9)	1.18 (1.01 to 1.37)
Eating disorder diagnosis assigned by specialized psychiatric care				
Any eating disorder	351 (63.8)	1407 (54.7)	9.0 (1.8 to 16.3)	1.11 (0.99 to 1.26)
Anorexia nervosa	145 (26.3)	557 (21.6)	4.7 (0.02 to 9.3)	1.20 (0.99 to 1.44)
Other eating disorders	265 (48.1)	1085 (42.2)	5.9 (-0.4 to 12.2)	1.07 (0.94 to 1.23)
Using attained age as underlying time scale				
Any eating disorder	389 (70.7)	1570 (61.1)	9.6 (1.9 to 17.2)	1.11 (0.99 to 1.24)
Anorexia nervosa	162 (29.4)	621 (24.1)	5.3 (0.4 to 10.2)	1.19 (1.00 to 1.42)
Other eating disorders	307 (55.7)	1254 (48.8)	7.0 (0.2 to 13.8)	1.08 (0.95 to 1.23)

	No. of events (IR, per 100,000 person-years)			
	Offspring of		IR difference (95%CI),	
	Patients	Reference individuals	per 100,000 person-years	HR (95%CI) [a]
Restricting to individuals without CeD				
Any eating disorder	254 (63.3)	1153 (58.5)	4.9 (-3.6 to 13.4)	1.05 (0.92 to 1.21)

© 2026 Yao J et al. *JAMA Network Open*.

Anorexia nervosa	88 (21.9)	469 (23.7)	-1.8 (-6.9 to 3.2)	0.89 (0.70 to 1.13)
Other eating disorders	211 (52.6)	911 (46.2)	6.4 (-1.3 to 14.1)	1.11 (0.95 to 1.29)
Restricting to individuals with available educational attainment data				
Any eating disorder	253 (65.7)	1023 (56.5)	9.2 (0.3 to 18.0)	1.06 (0.92 to 1.22)
Anorexia nervosa	91 (23.6)	392 (21.6)	2.0 (-3.3 to 7.2)	0.97 (0.77 to 1.23)
Other eating disorders	214 (55.5)	825 (45.6)	10.0 (1.9 to 18.0)	1.11 (0.95 to 1.29)
Excluding first one year of follow-up				
Any eating disorder	258 (62.2)	1112 (56.3)	5.9 (-2.4 to 14.1)	1.02 (0.89 to 1.18)
Anorexia nervosa	93 (22.4)	456 (23.0)	-0.7 (-5.7 to 4.3)	0.89 (0.70 to 1.12)
Other eating disorders	220 (53.0)	878 (44.5)	8.6 (1.0 to 16.1)	1.10 (0.95 to 1.28)
Excluding first three years of follow-up				
Any eating disorder	223 (54.7)	958 (49.5)	5.3 (-2.6 to 13.1)	1.03 (0.89 to 1.19)
Anorexia nervosa	80 (19.6)	399 (20.6)	-1.0 (-5.7 to 3.8)	0.87 (0.68 to 1.12)
Other eating disorders	193 (47.3)	759 (39.2)	8.2 (0.9 to 15.4)	1.12 (0.95 to 1.31)
≥2 diagnostic codes for eating disorders				
Any eating disorder	201 (48.3)	840 (42.4)	5.9 (-1.4 to 13.1)	1.05 (0.90 to 1.23)
Anorexia nervosa	76 (18.2)	348 (17.6)	0.7 (-3.8 to 5.2)	0.95 (0.73 to 1.23)
Other eating disorders	155 (37.2)	597 (30.1)	7.1 (0.8 to 13.4)	1.14 (0.95 to 1.36)
Eating disorder diagnosis assigned by specialized psychiatric care				
Any eating disorder	253 (60.8)	1032 (52.2)	8.7 (-0.6 to 16.8)	1.09 (0.94 to 1.25)
Anorexia nervosa	93 (22.3)	420 (21.2)	1.1 (-3.8 to 6.1)	0.97 (0.77 to 1.23)
Other eating disorders	203 (48.8)	782 (39.5)	9.3 (2.0 to 16.6)	1.14 (0.98 to 1.34)
Using attained age as underlying time scale				
Any eating disorder	277 (66.7)	1167 (59.0)	7.6 (-0.9 to 16.2)	1.07 (0.93 to 1.22)
Anorexia nervosa	101 (24.2)	471 (23.8)	0.5 (-4.7 to 5.7)	0.95 (0.76 to 1.18)
Other eating disorders	232 (55.8)	925 (46.7)	9.1 (1.3 to 16.8)	1.13 (0.97 to 1.30)

CeD: celiac disease; CI: confidence interval; bFDR: biological first-degree relative; HR: hazard ratio; IR: incidence rate

[a] adjusted for age, sex, county of residence, calendar year at index date, country of birth, educational attainment, number of healthcare visits between two years and six months before the index date, and separately for the history of type 1 diabetes, inflammatory bowel disease, and juvenile idiopathic arthritis (<16 years)/rheumatoid arthritis (≥16 years). Additionally, we adjusted for their index family members' age, sex, number of healthcare visits between two years and six months before the index date, and conditioned on a family identifier

eTable 17 Sensitivity analyses on eating disorders in bFDRs of patients with CeD before the index date, among individuals ≥6 years of age at their index family members' CeD diagnosis

	With/without eating disorders		
	Parents of		
	Patients	Reference individuals	OR (95%CI) [a]
Any eating disorder	142/68,979	566/326,123	1.04 (0.86 to 1.28)
Anorexia nervosa	55/69,066	208/326,481	1.05 (0.75 to 1.45)

© 2026 Yao J et al. *JAMA Network Open*.

Other eating disorders	113/69,008	440/326,249	1.08 (0.85 to 1.36)

	With/without eating disorders		
	Full siblings of		
	Patients	Reference individuals	OR (95%CI) [a]
Any eating disorder	236/45,299	950/210,404	1.05 (0.88 to 1.25)
Anorexia nervosa	89/45,446	356/210,998	0.89 (0.66 to 1.19)
Other eating disorders	180/45,355	749/210,605	1.05 (0.86 to 1.27)

	With/without eating disorders		
	Offspring of		
	Patients	Reference individuals	OR (95%CI) [a]
Any eating disorder	191/30,567	790/146,150	1.12 (0.94 to 1.35)
Anorexia nervosa	76/30,682	307/146,633	1.22 (0.90 to 1.65)
Other eating disorders	155/30,603	627/146,313	1.17 (0.95 to 1.43)

CeD: celiac disease; CI: confidence interval; bFDR: biological first-degree relative; OR: odds ratio

[a] conditioned on matching variables (age, sex, county of residence, and calendar year) and further adjusted for country of birth, educational attainment, number of healthcare visits between two years and six months before the index date, and separately for the history of type 1 diabetes, inflammatory bowel disease, and juvenile idiopathic arthritis (<16 years)/rheumatoid arthritis (≥16 years). Additionally, we adjusted for their index family members' age, sex, number of healthcare visits between two years and six months before the index date.

References

1. Subramanian L, Coo H, Jane A, et al. Celiac Disease and Inflammatory Bowel Disease Are Associated With Increased Risk of Eating Disorders: An Ontario Health Administrative Database Study. *Clin Transl Gastroenterol*. May 1 2024;15(5):e00700. doi:10.14309/ctg.0000000000000700

2. Hansen S, Osler M, Thysen SM, Rumessen JJ, Linneberg A, Kårhus LL. Celiac disease and risk of neuropsychiatric disorders: A nationwide cohort study. Nationwide cohort. *Acta Psychiatr Scand*. Jul 2023;148(1):60-70. doi:10.1111/acps.13554

3. Lebwohl B, Haggård L, Emilsson L, et al. Psychiatric Disorders in Patients With a Diagnosis of Celiac Disease During Childhood From 1973 to 2016. Nationwide cohort. *Clin Gastroenterol Hepatol*. Oct 2021;19(10):2093-2101.e13. doi:10.1016/j.cgh.2020.08.018 10.1016/j.cgh.2020.08.018. Epub 2020 Aug 12.

© 2026 Yao J et al. *JAMA Network Open*.

4. Alkhayyat M, Qapaja T, Aggarwal M, et al. Epidemiology and risk of psychiatric disorders among patients with celiac disease: A population-based national study. Population-based case control. *J Gastroenterol Hepatol*. Aug 2021;36(8):2165-2170. doi:10.1111/jgh.15437
10.1111/jgh.15437. Epub 2021 Feb 24.
5. Hedman A, Breithaupt L, Hübel C, et al. Bidirectional relationship between eating disorders and autoimmune diseases. *J Child Psychol Psychiatry*. Jul 2019;60(7):803-812. doi:10.1111/jcpp.12958
10.1111/jcpp.12958. Epub 2018 Sep 3.
6. Fasano A, Not T, Wang W, et al. Zonulin, a newly discovered modulator of intestinal permeability, and its expression in coeliac disease. *Lancet*. Apr 29 2000;355(9214):1518-9. doi:S0140-6736(00)02169-3 [pii]
10.1016/S0140-6736(00)02169-3
7. Mårild K, Størdal K, Bulik CM, et al. Celiac Disease and Anorexia Nervosa: A Nationwide Study. Nationwide cohort and case-control. *Pediatrics*. May 2017;139(5)doi:10.1542/peds.2016-4367
10.1542/peds.2016-4367. Epub 2017 Apr 3.
8. Butwicka A, Lichtenstein P, Frisén L, Almqvist C, Larsson H, Ludvigsson JF. Celiac Disease Is Associated with Childhood Psychiatric Disorders: A Population-Based Study. *J Pediatr*. May 2017;184:87-93.e1. doi:10.1016/j.jpeds.2017.01.043
10.1016/j.jpeds.2017.01.043. Epub 2017 Mar 7.
9. Wotton CJ, James A, Goldacre MJ. Coexistence of eating disorders and autoimmune diseases: Record linkage cohort study, UK. Nationwide cohort. *International Journal of Eating Disorders*. 2016;49(7):663-672. doi:10.1002/eat.22544
10. Nikniaz Z, Beheshti S, Abbasalizad Farhangi M, Nikniaz L. A systematic review and meta-analysis of the prevalence and odds of eating disorders in patients with celiac disease and vice-versa. **Systematic review and meta-analysis**. *Int J Eat Disord*. Sep 2021;54(9):1563-1574. doi:10.1002/eat.23561
10.1002/eat.23561. Epub 2021 May 27.
11. Ludvigsson JF, Brandt L, Montgomery SM, Granath F, Ekbom A. Validation study of villous atrophy and small intestinal inflammation in Swedish biopsy registers. *BMC Gastroenterol*. 2009-12-01 2009;9(1):19. doi:10.1186/1471-230x-9-19
12. Lebwohl B, Green PHR, Söderling J, Roelstraete B, Ludvigsson JF. Association Between Celiac Disease and Mortality Risk in a Swedish Population. Nationwide cohort. *JAMA*. Apr 7 2020;323(13):1277-1285. doi:10.1001/jama.2020.1943
10.1001/jama.2020.1943.
13. Miao J, Brismar K, Nyren O, Ugarph-Morawski A, Ye W. Elevated hip fracture risk in type 1 diabetic patients: a population-based cohort study in Sweden. *Diabetes Care*. Dec 2005;28(12):2850-5. doi:10.2337/diacare.28.12.2850

© 2026 Yao J et al. *JAMA Network Open*.
