# Supplementary material for: Eating Disorders in Individuals With Celiac Disease and Their Biological First-Degree Relatives
Source: JAMA Netw Open. 2026 Nov 12;9(11):e2643815. doi: 10.1001/jamanetworkopen.2026.43815 (PMC13760515; doi:10.1001/jamanetworkopen.2026.43815)
Supplement: Supplementary file 2 [file jamanetwopen-e2643815-s002.pdf]

# Data Sharing Statement

Yao. Eating Disorders in Celiac Disease and Biological First-Degree Relatives. *JAMA Netw Open*. Published November 12, 2026. doi:10.1001/jamanetworkopen.2026.43815

**Data**
**Data available:** No

**Additional Information**
**Explanation for why data not available:** The data set cannot be shared directly under current legislation for data protection and must be requested directly from the respective registry holders, Statistics Sweden (information@scb.se) and the Swedish National Board of Health and Welfare (registerservice@socialstyrelsen.se), after approval by the Swedish Ethical Review Authority.
